# Recent Advance in the Fabrication of 2D and 3D Metal Carbides-Based Nanomaterials for Energy and Environmental Applications

**DOI:** 10.3390/nano11010246

**Published:** 2021-01-18

**Authors:** Keming Wan, Yalin Li, Yan Wang, Gang Wei

**Affiliations:** College of Chemistry and Chemical Engineering, Qingdao University, Qingdao 266071, China; wkm17853243755@163.com (K.W.); liyalinzl0919@163.com (Y.L.)

**Keywords:** metal carbides, two-dimensional materials, three-dimensional, hybrid nanomaterials, energy storage, environmental science

## Abstract

Two-dimensional (2D) nanomaterials have attracted increased interest and exhibited extended applications from nanotechnology to materials science, biomedicine, tissue engineering, as well as energy storage and environmental science. With the development of the synthesis and fabrication of 2D materials, a new family of 2D materials, metal carbides (MCs), revealed promising applications in recent years, and have been utilized for the fabrication of various functional 2D and three-dimensional (3D) nanomaterials for energy and environmental applications, ascribing to the unique physical and chemical properties of MCs. In this review, we present recent advance in the synthesis, fabrication, and applications of 2D and 3D MC-based nanomaterials. For this aim, we first summarize typical synthesis methods of MCs, and then demonstrate the progress on the fabrication of 2D and 3D MC-based nanomaterials. To the end, the applications of MC-based 2D and 3D materials for chemical batteries, supercapacitors, water splitting, photodegradation, removal of heavy metals, and electromagnetic shielding are introduced and discussed. This work provides useful information on the preparation, hybridization, structural tailoring, and applications of MC-based materials, and is expected to inspire the design and fabrication of novel and functional MXene materials with improved performance.

## 1. Introduction

Two-dimensional (2D) materials such as graphene, transition metal oxides (TMO), transition metal dichalcogenides (TMD), and other have been widely studied in the last few decades [1,2,3]. Compared to transitional zero-dimensional (0D) and one-dimensional (1D) nanomaterials, 2D materials exhibited unique properties and advantages due to their atomic layer thickness, large surface area, high electrical conductivity, strong mechanical strength, and flat nano/micro-scale surface, which making them excellent candidates for the design and fabrication of multifunctional nanomaterials towards applications in energy science, [4,5] environmental science, [6] electronic devices, [7] sensors, [8,9] tissue engineering, [10] biomedicine, [11] and many others. Recently, more and more attentions have been attracted onto one of the members of 2D materials family, MXenes, which include typical emerging class of 2D transition metal carbides (MCs) and metal nitrides (MNs) [12,13,14]. It has been found that 2D MCs and MNs have similar physical, chemical, and biological properties to that of graphene-like 2D materials. In addition, MCs and MNs have the surface functional groups of -O, -OH, and -F, as well as the elements of C or N, making them not only easy to modify with surface chemistry, but also high potential as electrode materials in catalysis, batteries, supercapacitors, and sensors [15,16,17,18].

To improve the functionalities of MCs and extend their applications, various strategies have been utilized to tailor the properties, hybridization, and structures of MC-based materials. For instance, the surface functionalization of MC with -F, -OH, and -O groups has been proved to an effective way to create novel electronic and magnetic properties of 2D transition MCs and -MNs [19]. Wang et al. demonstrated the modification of titanium carbide (Ti_3_C_2_) with chimeric peptides, which could be served as a good biosensor platform for detecting the activity of enzymes [20]. Besides the surface, MCs can be hybridized with other nanoscale building blocks such as nanoparticles [21], quantum dots [22], carbon materials [23], and others to enhance the material properties through synergistic effects. In addition, the functional tailoring of MC-based nanomaterials could be achieved by adjusting the structures of materials from 2D nanosheets, films, and membranes to 3D scaffolds, hydrogels, and aerogels [24,25,26]. Previously, great achievements on the synthesis, functionalization, and applications of various MC-based nanomaterials, and a lot of very important reviews have been released [27,28,29,30,31,32]. For instance, Yan et al. presented a comprehensive review on the synthesis of nanostructured metallic transition metal carbides, nitrides, phosphides, and borides for the applications in energy storage and conversion, in which more attentions were focused on developing new strategies to improve electrochemical performances of nanostructured 2D materials [28]. Li and Wu summarized recent progress of 2D transition MCs and their nanocomposites for the applications of photo/electrocatalysis and conventional heterogenous catalysis ascribed to the adjusting of the chemical, electronic, and mechanical properties of MCs [30]. Very recently, Shahzad and co-workers demonstrated recent advance in the using of 2D transition MCs for electrochemical sensing of diverse types of heavy metals, dyes, small molecules, and biomolecules as well as biomarkers [32]. 

**Scheme 1:**
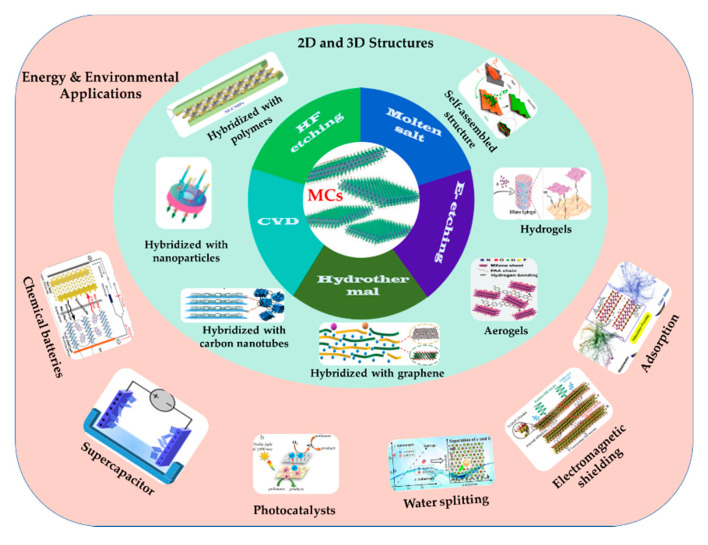
Fabrication, structures and applications of various metal carbides-based nanomaterials.

By studying the above literatures, we found that it is necessary to contribute additional information on the fabrication of 2D and 3D MC-based materials by different techniques and strategies, which will provide effective guiding for readers to understand the fabrication of functional MC materials for advanced applications. In addition, the presentation of MC-based 2D and 3D materials for energy and environmental applications will inspire the design and preparation of novel MXene materials beyond MCs and promote their applications in materials science, nanotechnology, tissue engineering, biomedical engineering, and other corresponding fields. For this aim, in this work we summarize recent progress on the synthesis, structural fabrication, hybridization, as well as energy and environmental applications of MC-based 2D and 3D nanomaterials, and the main contents are shown in Scheme 1.

## 2. Fabrication Methods of 2D MC MXene.

Generally, there are two ways to synthesize 2D MXene materials. The first one is the bottom-up approach, such as the chemical vapor deposition (CVD) to produce high-quality films on various substrates. The second one is the top-down method, which involves the liquid-phase peeling and ball milling method. In this section, we focus on the synthesis of MC MXenes by both bottom-up and top-down techniques, including CVD, hydrofluoric acid (HFA) etching, molten salt synthesis, electrochemical etching, and hydrothermal approach as these methods have been proved to be effective to fabricate MXene [33].

### 2.1. CVD

CVD is known as a typical bottom-up strategy. The principle of CVD is to introduce one or more gaseous substances into a reaction chamber while undergoing a chemical reaction, where a new material is deposited on the surface of the substrate. It is currently the most widely used technology for large-scale industrial preparation of thin film materials. Among various thin film materials, the synthesis of MC MXenes that employs CVD approach has been extensively reported. For example, Xu et al. [34] reported a method for preparing large-area high-quality 2D ultra-thin α-Mo_2_C crystals by chemical vapor deposition. The as-synthesized crystal was a few nanometers thick with a size of more than 100 µm, and was very stable under environmental conditions. It is important that the developed CVD method is universal and can be adopted as a general strategy to manufacture a wide range of high-quality 2D ultrathin 2D MXene crystals, such as ultrathin WC and TaC crystals, which further expands the range of 2D materials. Wang et al. [35] proved that the CVD method can also be used to grow 2D ultrathin Ta-based compounds by heating a Ta-Cu bilayer with corresponding precursor. DFT calculations further confirmed this approach. With the help of different precursors (gas or powder), temperature and time adjustment can control the diffusion of Ta clusters in Cu, thereby converting immiscible clusters into different ultrathin carbides, nitrides and borides. The chemical vapor deposition method can meet the requirements of large-scale production of high-quality, large-area MXene. However, due to its relatively high cost, complicated technology and precise control of processing conditions, the development of CVD for preparing MXene is restricted, and further research on exploring facile and cost-effective techniques for the fabrication of MXenes is needed. 

### 2.2. HFA Etching

HFA etching is currently the most commonly approach for the synthesize of MCs. In fact, most MCs can be obtained by selectively etching the A layer from the MAX phase by controlling the reaction time and HFA concentration at low temperatures (usually lower than 558 °C) [36], because the M-A bonds are relatively weak and easy to break compared with the M-X bonds [37]. For example, taking aluminum carbide as an example, the synthesis of MCs could be achieved by etching an atomic layer of element A (e.g., aluminum) in the MAX phase (e.g., Ti_3_AlC_2_) from the M_n+1_Xn layer adjacent to the element A layer [38].

**Figure 1:**
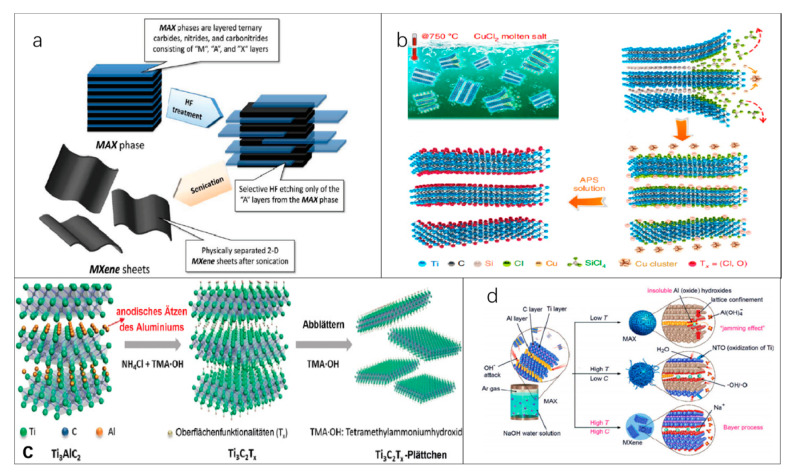
(**a**) The etching process of MAX phase and the formation of MXene. Reproduced from [38], with permission from, American Chemistry Society, 2012. (**b**) Schematic of MS-Ti_3_C_2_T_x_ MXene preparation process. Reproduced from [39], with permission from Nature Publishing Group, 2020. (**c**) Electrochemical method of Ti_3_C_2_ stratification in binary water electrolyte. Reproduced from [40], with permission from Wiley, 2018. (**d**) The preparation of Ti_3_AlC_2_ using different temperatures and concentrations of sodium hydroxide, where higher temperatures and alkali concentrations help dissolve Al, and push the entire etching process to the production of MXene. Reproduced from [41], with permission from Wiley, 2018.

Naguib et al. [38] firstly reported the synthesis of 2D transition MCs and metal carbonitrides by select immersing MAX phase powders in the HFA solution. They first attempted to exfoliate the MAX phases by immersing Ti_3_AlC_2_ powders in 50% HFA at room temperature for 2h. This procedure gave rise to the selective etching of the Al layers and their replacement by OH- and S surface groups. Besides, they supposed that Ti_3_C_2_ can represent a member of the family of 2D transition metal carbides and/or nitrides very well. The stripping process of MAX phase and the formation of MXenes are shown in Figure 1a.

Since the first attempt of employing HFA to etch Ti_3_AlC_2_ for the synthesis of multilayer Ti_3_C_2_T_x_ in 2011, the HFA etching method has gradually been accepted and adopted. However, HFA is a corrosive chemical substance that can penetrate the skin, muscle tissue and bones, causing handling and disposal hazards. In recent years, the avoidance or minimization of the use of concentrated HFA during MC synthesis has become a research trend. An alternative way is the utilization of hydrochloric acid (HCl) and fluoride salt to form HFA. For example, Ghidiu et al. [42] developed a safer way by using the reaction between ordinary cheap HCl and lithium fluoride (LiF), which leads to minimal dissolution of aluminum and extraction of the 2D carbide layer. The MXene was prepared by dissolving LiF in 6 M HCl, then slowly adding Ti_3_AlC_2_ powder, and heating the mixture at 40 °C for 45 h. After etching, the resulting precipitate was washed to remove the reaction product and the pH value was increased by adding water and then centrifugating and decanting for several cycles. In fact, LiF and HCl corrosion is milder and multiplexer than HFA, and the resultant MCs exhibit more lateral size expansion, excluding the nanoscale defects that are often observed in HFA corrosion samples. Thus, by employing this approach, a large number of single-layered MC flakes can be easily produced, which present high yields, large lateral sizes and satisfied quality. In another case, Guan et al. [43] reported a HFA-free synthesis of 2D vanadium carbide (V_2_C) MXene by mild etching V_2_AlC powders in the mixture of LiF and HCl. The synthesized V_2_C MXene after 120 h of etching presented a uniform multilayer structure and a high purity (>90%). This method does not form any by-products during the synthesis of V_2_C MXene. Consequently, the as-prepared V_2_C MXene was used as the electrode material for the fabrication of supercapacitors with good performance. 

In general, both HFA and HCl/LiF etching are the most mature synthetic routes for layered carbides and carbonitrides [44]. Each method produces multilayer MXene with different morphologies and surface chemistry. Unfortunately, both etching methods would cause environment pollution due to the presence of fluorine. At present, more environmentally friendly and efficient methods for synthesizing MXene are being continuously explored.

### 2.3. Molten Salt Method

Because fluorine-containing acidic solutions are dangerous to human beings, researchers continue to seek alternative MXene synthesis techniques [45]. Among various HFA-free methods, molten salt method, as a relatively low-temperature nanocarbide synthesis technology, has attracted more and more attention in recent years. The method uses salt as a liquid medium, and the reaction is faster at a lower temperature, and the reaction completion time is shorter. So far, the molten salt method has been utilized successfully for the synthesis of Ti_3_SiC_2_ and Cr_2_AlC. Unfortunately, the particle size of the largest phase prepared is also larger than a few microns. Therefore, there is an urgent need to reduce the grain size of the obtained Ti_3_AlC_2_ powder to promote faster conversion of Ti_3_AlC_2_ into 2D Ti_3_C_2_. Recently, a researcher has successfully synthesized fine-grained Ti_3_AlC_2_ powder with a salt/precursor material (titanium, aluminum, carbon powder) ratio of 10:1 at 950 °C for 5 h [46]. The nanoscale Ti_3_AlC_2_ powder that synthesized by the molten salt method can convert Ti_3_AlC_2_ into 2D Ti_3_C_2_ faster. On this basis, Ding et al. [47] allowed Ti_2_AlN and Nb_2_AlC to perform A-site replacement reactions with CuCl_2_ and CuI molten salts, respectively, and synthesized new Ti_2_(Al_0.1_Cu_0.9_)N and Nb_2_CuC MAX phases. Next, density functional theory (DFT) calculations were used to verify the structural stability of these new MAX phases and predict their elasticity and the low cleavage energy of the M-A bond. These results have also inspired more researchers to explore strategies for preparing MXene with this environmentally friendly, non-toxic molten salt. For example, Li et al. [48] demonstrated a common approach to synthesize a series of Zn-based MAX phases and Cl-terminated MXene deriving from the replacement reaction between the MAX phase and the late transition-metal halides. This approach is known as a typical top-down route, which allows the late transitional element atom, i.e., Zn, to take up the A site in the pre-existing MAX phase structure. Novel MAX phases Ti_3_ZnC_2_, Ti_2_ZnC, Ti_2_ZnN and V_2_ZnC can be synthesized by employing the replacement reaction between the Zn element of molten ZnCl_2_ and the Al element in MAX phase precursors (Ti_3_AlC_2_, Ti_2_AlC, Ti_2_AlN, and V_2_AlC). When an excessive amount of ZnCl_2_ is used, Cl-terminated MXene such as Ti_3_C_2_Cl_2_ and Ti_2_CCl_2_can be produced subsequently by the exfoliation of Ti_3_ZnC_2_ and Ti_2_ZnC since the molten ZnCl_2_ holds strong Lewis acidity. 

In order to continue to expand the application of MXene materials, on the basis of the above melting method, Lewis acid salt melting method has been developed. Recently, Li and co-workers proposed a generic approach to etch MAX phases by direct redox-coupling between the A element and the cation of the Lewis acid molten salt [39]. Figure 1b presents the synthesis of the Ti_3_C_2_T_x_ MXene through the reaction of Ti_3_SiC_2_ and CuCl_2_. The Ti_3_SiC_2_ MAX precursor was immersed in molten CuCl_2_ at 750 °C. The Si atoms that are exposed to the Ti_3_C_2_ sub-layer weakly bonded to Ti are oxidized by Lewis acid Cu^2+^ to Si^4+^ cations, forming a volatile SiCl_4_ phase and accompanying reduction of Cu^2+^ to Cu metal. Excess Cu^2+^ reacts with Ti atoms exposed to Ti_3_C_2_ to form metallic Cu, simultaneously, Ti_3_C_2_Cl_2_ was formed by Cl-anion reaction to ensure equal charge. The formation mechanism of Ti_3_C_2_Cl_2_ from Ti_3_SiC_2_ is similar to that of chemically etching Ti_3_AlC_2_ in HF solution. Subsequently, Cu particles were removed from the surface of Ti_3_C_2_Cl_2_ MXene by immersing the prepared Ti_3_C_2_Cl_2_ and Cu powders in ammonium persulfate (APS) solution, which also resulted in the addition of O-based surface groups (Figure 1b). The resultant material synthesized by this molten salt route was called MS-Ti_3_C_2_T_x_ MXene, in which T_x_ denotes O and Cl surface groups and MS stands for molten salt. The as-prepared MXene exhibited much improved electrochemical performance, excellent storage capability as well as high-rate performance in non-aqueous electrolytes. These excellent properties endowed the synthesized Ti_3_C_2_Tx MXenes to be used in high-rate batteries and hybrid devices, such as lithium-ion batteries and supercapacitors.

### 2.4. Electrochemical Etching

It is well known that the end groups of MXene materials are very rich, but some end groups (F^−^ terminated groups) materials limit its application in adsorption, capacitor, electromagnetic shielding, and others [49]. At present, the development of electrochemical etching is expected to solve this problem. Electrochemical etching is a more environmentally friendly, effective, fluorine-free electrochemical method, which is generally used to layered Mxene in binary aqueous electrolytes. Taking Ti_2_AlC as an example, since the Ti-Al bond is weaker than the Ti-C bond, the electrochemical etching of Ti_2_AlC with Cl etchant is usually divided into two processes. In the first step, the applied voltage first removes Al atoms from the layered carbide; then in the second stage, both aluminum and titanium atoms are etched until only one layer of carbon atoms remains.

For instance, Pang et al. [33] proposed a thermal-assisted electrochemical etching method to prepare HFA-free MXenes, i.e., Ti_2_CT_x_, Cr_2_CT_x_, and V_2_CT_x_, using a 3D composite electrode with carbon fiber cloth (CFC) porous substrate and carbon black (CB) additives. In the presence of dilute HFA, gentle heating accelerated the etching of MAX material. Their work paved a way to develop HFA-free and rapid synthesis of 2D layered MXene targeting for effective absorption of heavy metals and multifunctional electrocatalysis and energy storage applications. Recently, Yang et al. [40] demonstrated an effective strategy to prepare Ti_3_C_2_T_x_ (T = O, OH) MXene using electrochemical etching, as shown in Figure 1c. The electrochemical etching mechanism can be explained as follows:

Ti_3_AlC_2_ +3Cl^−^ → Ti_3_C_2_ + AlCl_3_ + 3e^−^ (1)

Ti_3_C_2_ + 2OH → Ti_3_C_2_(OH)_2_ + 2e^−^ (2)

Ti_3_C_2_ +2H_2_O → Ti_3_C_2_(OH)_2_ + H^−^ (3)

Reaction (1) is essential to the aluminum layer from the parent Ti_3_AlC_2_ anode. Then, reactions (2) and (3) show that the Ti_3_C_2_ surface is OH^−^ terminated. The Ti_3_AlC_2_ can be modeled as a layer of aluminum atoms with two layers of Ti_3_C_2_ sandwiched between them (Figure 1c). When Ti_3_AlC_2_ is positively charged, it starts to attack the edge of Ti_3_AlC_2_. Subsequently, the dissociation of the Al atoms leads to the formation of AlCl_3_ and terminates the edge Ti atoms with Cl^−^. The further opening of the grain boundary promotes the further penetration of NH_4_OH and Cl^−^, which will extract aluminum atoms and weaken the Ti-Al bond. The hydroxyl functional group on the Ti_3_C_2_ layer is formed by the nucleophilic attack of the surrounding OH^−^. Since the OH^−^ radius (0.11 nm) is smaller than the Cl^−^ radius (0.18 nm), it is easier to insert and erode the surface of the Ti_3_C_2_ sheet. In this approach, chloride ion can corrode aluminum quickly and destroy Ti-Al bond. The subsequent intercalation of ammonium hydroxide (NH_4_OH) helps to open the edges of the etched anode, which enables etching underlying the surfaces. The anode (Ti_3_AlC_2_) is quickly etched under ambient conditions. More than 90% of the stripped materials obtained by this method are single-layer or double-layer, and the average lateral size exceeds 2 m, outperforming the size obtained by the classic HF etching process. Besides, there is no fluorine terminations in the exfoliated Ti_3_C_2_T_x_ due to the fluoride free etching. The additive-free film has excellent surface capacitance (220 mF cm^−2^) for the application in super capacitors, which is better than that produced by traditional wet chemical methods.

However, over-etching of parent MAX phases to carbide-derived carbon (CDC) often happens during electrochemical etching. To avoid this phenomenon, Sun et al. [50] developed a core-shell model to explain electrochemical etching of Ti_2_AlC to Ti_2_CT_x_ and CDC. The proposed model suggests that a careful balance in etching parameters is required to produce MXenes while avoiding over-etching. Subsequently, they etched Al with a porous Ti_2_AlC electrode in dilute HFA by electrochemical method, and obtained a Ti_2_CT_x_ MXene layer that formed on Ti_2_AlC successfully. This electrochemical method expands the possible range of etching techniques and the final composition of MXene.

### 2.5. Hydrothermal Method

With the development of 2D material synthesis technology, the application of MXene materials is more and more extensive, and the preparation requirements are gradually increasing. However, the development of new manufacturing processes is also a challenge [51,52]. In order to meet this challenge, Li et al. [41] used alkali-assisted hydrothermal approach to synthesize MXene Ti_3_C_2_T_x_ with the purity of 92 wt% by using 27.5 M NaOH at 270 °C. Without F termination, the as-prepared Ti_3_C_2_T_x_ film performed better than the multilayer Ti_3_C_2_T_x_ produced by HFA treatment. Such the fluorine-free method gives an alkali etching strategy for the development of new MXene. In the preparation process of Ti_3_AlC_2_, OH¯ attacking the Al layer can be roughly divided into two steps. In the first step, Al was oxidized into aluminum hydroxide. In the second step, aluminum hydroxide was dissolved in alkali. Figure 1d shows different temperatures and concentrations of sodium hydroxide to etch MXene materials, and higher temperature and alkali concentration help dissolve Al (oxidized) hydroxide. This method is very suitable for removing aluminum compounds and to push the entire etching process toward the production of MC MXene [33].

In order to study the influence of temperature and alkali concentration on the etching method, Wang et al. [53] described a mild, simple one-pot hydrothermal method to prepare Ti_3_C_2_T_x_ without using HFA. In this approach, the growth of Ti_3_C_2_Tx nanomaterials was affected by the temperature, the concentration of NH_4_F, and the processing time. The X-ray diffraction (XRD) analysis showed that higher temperature and NH_4_F concentration resulted in higher yield of Ti_3_C_2_T_x_. It was a safe and simple method to prepare high-performance Ti_3_C_2_T_x_ by NH_4_F hydrothermal synthesis. In addition, Ti_3_C_2_T_x_ synthesized by this method has exhibited excellent capacitance performance. In the process of producing supercapacitor electrodes, Ti_3_C_2_T_x_ synthesized by NH_4_F hydrothermal method also showed the ability to provide fast electron transfer channels and increase large electrochemically active surfaces [53].

The hydrothermal method is a simple, green method that avoids the direct use of hydrogen fluoride or fluoride. For 2D MXene, the development of this method has become particularly important, especially as energy storage materials. Recently, one novel approach has been proposed by Peng et al. [54] for the synthesis of Ti_3_C_2_ MXene (H-Ti_3_C_2_) through the hydrothermal method to prepare low-toxic etching agents (NaBF_4_, HCl). The as-prepared h-Ti_3_C_2_ has the higher c lattice parameter, larger interlayer distance and larger specific surface area, comparing to the Ti_3_C_2_ produced by the traditional HFA etching method(t-Ti_3_C_2_), primarily owing to the slow release during the hydrothermal process. The hydrothermal etching method not only avoids the use of high concentrations of HFA, but also prepares Ti_3_C_2_ flakes with high efficiency. Moreover, the hydrothermal etching route can be extended to synthesize other MXene materials. Their work provided a versatile, safe and efficient method for the synthesis of 2D MXene materials.

### 2.6. Summary

To make it clearer, we summarize the above-mentioned synthesis methods of MC MXenes including the advantages and drawbacks of each method, representative MC MXenes, and their challenges in fabrication process, as shown in in Table 1.

## 3. 2D and 3D Structures of MXene-Based Nanomaterials

In this part, the latest developments in the 2D structure and 3D structures based on Mxene at the macro and micro levels are introduced, and the assembly mechanisms are discussed to better understand the relationships between the performance and formed structures [55].

### 3.1. 2D Structures

#### 3.1.1. Hybridized with Polymers

2D MC MXene has received widespread attention owing to its distinct physical and chemical properties. Nevertheless, MC nanosheets are easily affected by heavy pressure and oxidation, thereby losing their functional features and limiting their applications [56]. Therefore, it is advisable to explore strategies to prevent overlap of MC nanosheets and avoid oxidation. Conjugated microporous polymers (CMPs) are known as a category of organic porous materials, which are composed of rigid conjugated molecules through covalent bonds with a 3D network framework and intrinsic porous structure. Due to its facile and cost-effective synthesis large specific surface area and tunable network structure as compared to traditional polymers, CMP has gained extensive attention especially in the field of energy storage. Recent studies have shown that adding CMP between MC nanosheets can effectively inhibit the accumulation of MC and enlarge the layer spacing, and it can also prevent the surface from being oxidized.

**Figure 2:**
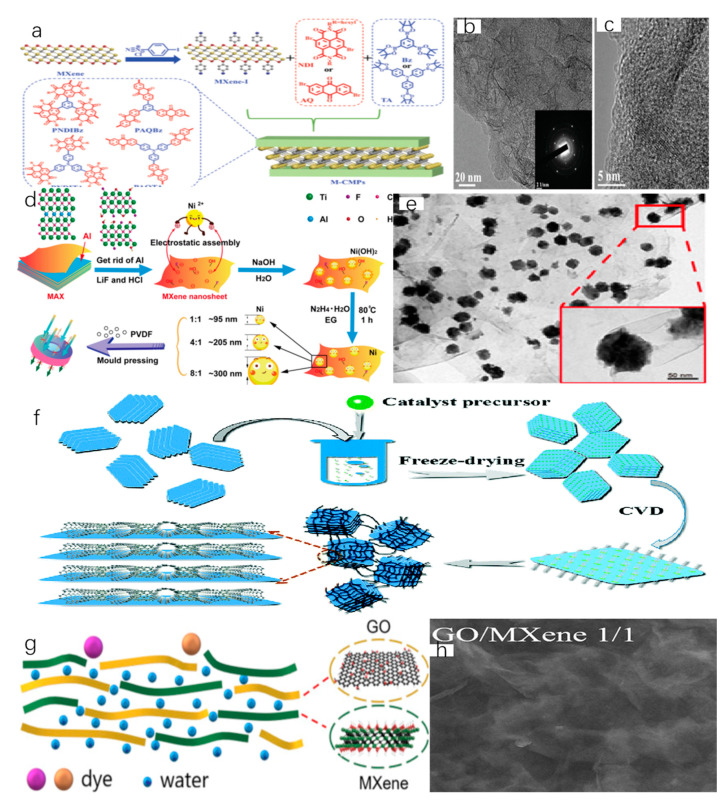
(**a**) Schematic of the M-CMP synthesis program. (**b**) Area electron diffraction pattern of M-CMP nanosheet. M-CMP still preserves an apparent relatively obvious sheet structure, indicating that the reaction did not cause serious accumulation and thus destroy the structure of MXene. (**c**) High resolution TEM (HRTEM) image of M-CMP’s microporous structure. The light and dark parts are alternately displayed, which can explain the micropore structure with uniform pore distribution in M-CMP. Reproduced from [57], with permission from Wiley, 2020. (**d**) The synthesis method and formation mechanism of Ni@MXene heterides. (**e**) TEM image of Ni@MXene hybrid, in which Ni nanoparticles are uniformly attached to the Ti3C2Tx nanosheets. Reproduced from [58], with permission from American Chemical Society, 2019. (**f**) Schematic diagram of Ti_3_C_2_T_x_/CNT complex synthesized by CVD. Reproduced from [59], with permission from Royal Society of Chemistry, 2017. (**g**) Mechanism of GO/MXene composite membrane in water purification process. (**h**) SEM images on the surface of GO/MXene composite film with mass ratio of 1/1. Reproduced from [60], with permission from Elsevier, 2019.

Recently, Yang et al. [57] demonstrated an effective strategy to produce MC-based conjugated microporous polymer (M-CMP) by forming sandwiched structure of MXene and CMPs, as shown in Figure 2a. The prepared M-CMP has a hierarchical porous structure of MXene and a large specific surface area, while inhibiting the overlap and oxidation of MXene, and has a 2D structure and high conductivity. These outstanding structural advantages are the key to electrochemical energy storage. Subsequently, they observed the morphology of M-CMP through scanning electron microscopy (SEM). Compared with the amorphous and random agglomeration morphology of pure polymer, all M-CMP exhibited a clear sheet structure. M-CMP has a rigid 2D flake morphology with a lateral dimension of several microns, which is analogous to the morphology of the original MXene nano flakes. However, unlike the smooth surface of MXene, the surface of the M-CMP composite presents nearly identical roughness as free CMP due to the irregular porous CMP encapsulated on MXene. In addition, no pure CMP or “bare” MXene nanosheets can be found, indicating that the majority of polymer units have been fixed on the MXene surface. Figure 2b,c show a further study of the distribution of CMP anchored on MXene. The transmission electron microscopy (TEM) image of M-CMP indicates that after MXene and CMP are covalently connected, M-CMP still preserves an apparent sheet structure, which indicates that the reaction does not cause serious accumulation of MXene, and thus does not destroy the structure of MXene (Figure 2b). The corresponding selected area electron diffraction pattern (set in Figure 2b) of the M-CMP nanosheet clearly shows the hexagonal structure, indicating that MXene and CMP are arranged in a sandwich configuration. In addition, the microporous structure with uniform pore distribution in M-CMP can be explained by the alternate display of light and dark parts in the high-resolution TEM (HRTEM) image (Figure 2c). Such results indicate that no significant stacking of MXene nanosheets happened during the polymerization process. The researchers also found that the morphology of M-CMP composites can be customized by changing the loading of CMP on MXene. This can be achieved by changing the weight ratio of CMP to MXene (8:5, 8:10, 8:15, and 8:20) to realize. It is foreseeable that these composite materials formed by covalently connecting MXene and CMP effectively extend the interlayer distance of MXene on one hand, and increase the available active sites of CMP and reduce the ion diffusion path on the other hand, thus being used as supercapacitor electrodes [57].

It should be pointed out that most MXenes are unstable in long-term operation, which is a potential challenge to overcome. One possible solution is to post-process the MXene flakes before mixing with the corresponding polymers. The function of MXene surface has a great influence on its performance, and morphological modification is also very important for the increase of active sites. Surface modification of MXene is not only beneficial to obtain MXene with ideal properties, but also facilitate to increase the interaction between MXene flakes and polymer chains. Therefore, the stability and service life of the MXene/polymer film can be improved.

#### 3.1.2. Hybridized with Nanoparticles

Unparalleled conductivity makes MXene and its derivatives show huge potential in lithium-ion batteries, water treatment, sensors, supercapacitors, electromagnetic wave absorption and shielding. However, pure Ti_3_C_2_T_x_ MXene usually leads to unbalanced electromagnetic (EM) parameters and cannot achieve good impedance matching, so it is not suitable for direct use as adsorption and electromagnetic shielding materials. Nanoparticles are artificially manufactured micro-particles with a size of no more than 100 nm. Its form may be latex, polymer, ceramic particles, metal particles and carbon particles. Nanoparticles often have a large specific surface area. In recent years, they have showed important applications in fillers, medicine, magnetism, and catalysis. In magnetic applications, nanoparticles have the characteristics of high magnetic saturation, high temperature resistance, and strong corrosion resistance, and they are considered as potential electromagnetic shielding objects. Therefore, combining the magnetic loss of magnetic nanoparticles and the dielectric loss of 2D Ti_3_C_2_T_x_ MXene can not only theoretically improve impedance matching and effectively attenuate electromagnetic waves, but also quickly release the converted heat energy through MXene nanosheets [61]. Moreover, the in-situ growth of magnetic metal nanoparticles on MXene can further improve its interfacial interaction capability with enhanced synergistic loss effect, which suggests great application potential of the MXene

At present, one method for synthesizing layered Ti_3_C_2_T_x_ MXene hybrids anchored by magnetic metal nanoparticles is to use mild and size-controllable co-solvents [58]. The synthesis method and formation mechanism of Ni@MXene hybrid are shown in Figure 2d. Because the surface of Ti_3_C_2_T_x_ nanosheets is negatively charged, Ni^2+^ are attached to Ti_3_C_2_T_x_ nanosheets by electrostatic adsorption, which provides homogeneous and effective nucleation sites for the growth of Ni nanoparticles. Then, the connected Ni^2+^ complexes with OH^−^ forms a precursor of Ni(OH)_2_ under alkaline conditions_._ Finally, the formed Ni(OH)_2_ is reduced to a metallic Ni core. Through repeated attachment and reduction, Ni nanoparticles were produced and then immobilized on Ti_3_C_2_T_x_ nanosheets to obtain the Ni@MXene hybrids. The microstructure and morphology of the as-synthesized Ni@MXene hybrids were characterized subsequently by [58] SEM and TEM techniques. It was found that the existence of negatively charged Ti_3_C_2_T_x_ nanosheets can not only serve as a nucleation carrier for Ni growth, but also prevent Ni from agglomerating, which eventually leads to uniform size and distribution of Ni nanoparticles on Ti_3_C_2_T_x_ as confirmed by the TEM image (Figure 2e). Further calculations show that due to the large addition of nickel, the size of nickel nanoparticles has increased from 95 nm (Ni@MXene, 1: 1) and 205 nm (Ni @MXene, 4: 1) to 300 nm (Ni@MXene, 8: 1). This indicates that the content and size of Ni nanoparticles gradually increase with increasing Ni^2+^. When the mass ratio is relatively low (at 1:1 and 4:1), Ni nanoparticles exhibit a homogeneous distribution on the surface of MXene nanosheets. Whereas for a high mass ratio of 8:1, some Ni agglomerations will occur due to too much nickel to be reduced. After about 1 h of ultrasonic TEM measurement, Ni nanoparticles are still stably anchored on MXene, implying a strong interfacial adhesion effect between Ni nanoparticles and MXene. The uniform dispersion and strong adhesion of Ni nanoparticles on Ti_3_C_2_T_x_ nanosheets facilitate to absorb EM waves fully and effectively, showing the potential of electromagnetic shielding.

#### 3.1.3. Hybridized with Carbon Nanotubes

As a 1D nanomaterial, carbon nanotubes (CNTs) are light in weight, perfectly connected in hexagonal structure, and have many unusual mechanical, electrical and chemical properties. In recent years, with the deepening of research on CNTs and nanomaterials, its broad application prospects have also been constantly revealed. As mentioned above, Ti_3_C_2_T_x_ MXenes has good design of chemical and physical properties due to its customizable surface structure, such as active surface, functional groups, and local hierarchical structure. In the above part, we described that nanoparticles could be attached uniformly to Ti_3_C_2_T_x_ and inserted into the interlayer gap through reasonable interface contact to improve the electromagnetic shielding effect. At present, researchers have found that combining 1D high-dielectric loss CNTs and 2D low-dielectric loss Ti_3_C_2_T_x_ MXenes to form a Ti_3_C_2_T_x_/CNT hybrid 2D material is another effective method to improve the electromagnetic wave absorption performance of the material [62].

Li et al. [59] used a simple catalytic CVD process to prepare in-situ CNT-modified Ti_3_C_2_T_x_ MXenes (Figure 2f). The prepared Ti_3_C_2_T_x_/CNTs nanocomposite material shows that 1D CNTs are uniformly distributed between the layers of 2D Ti_3_C_2_T_x_ MXenes sheets, forming a layered sandwich microstructure and contributing excellent electromagnetic wave absorption performance. As shown in Figure 2f, CNTs were evenly distributed in the middle layer of the Ti_3_C_2_T_x_ MXene sheet, and the obtained Ti_3_C_2_T_x_ MXenes had a typical multilayer morphology. This can be attributed to the uniform dispersion of the catalyst precursor on one hand, and the porous microstructure inside the Ti_3_C_2_T_x_ particles to offer sufficient space for the growth of CNTs on the other hand. Further study indicated that Ti_3_C_2_T_x_ particles were bridged by curved nanotubes and gradually cross-linked into the entire nanoporous network. Interestingly, Ti_3_C_2_T_x_ particles still preserved their multilayered microstructure after CVD growth, facilitating EM wave dissipation. Moreover, CNTs were evenly distributed on the surface of the Ti_3_C_2_T_x_ MXene sheet, connecting the Ti_3_C_2_T_x_ to form the entire network. This layered network structure has two important functions in electromagnetic shielding. Firstly, the connecting effect of CNTs with high conductivity can offer more conductive paths for charge carriers, which reduces the loss of conductivity. Secondly, the produced huge area of interfaces including Ti_3_C_2_T_x_/CNT and CNT/CNT interfaces are very beneficial for interface polarization. When charge carriers migrate to heterogeneous interfaces, nanoparticle boundaries, residual functional groups and other locations (such as vacancies in CNTs, dislocations and dangling bonds), a portion of the unneutralized charges will accumulate in these locations, which forms a large number of dipoles and greatly increases the loss of polarization. Therefore, by introducing CNTs, the loss of conductivity and the establishment of various polarizations can dissipate a large amount of EM wave energy [59].

#### 3.1.4. Hybridized with Graphene

Among 2D materials, the research on graphene oxide (GO) and MXene is more attractive [63]. The GO material also contains a large number of oxygen-containing groups, such as hydroxyl, carboxyl and epoxy groups, which are located on the edge and base plane of GO nanosheet. These functional groups on GO nanosheets facilitate the dispersion of GO in water and enhance the interaction with water molecules and ions during water purification. The strong interaction between functional groups and separable pollutants endow GO membranes excellent separation performance, unfortunately, the low water permeability induced by the formation of hydrogen bonds between GO and functional groups on water molecules is still an ongoing issue. MXenes, as a new series of 2D materials based on transition MCs and/orMNs, has been recently adopted for water remediation due to their complex and diverse structure as well as superior mechanical, physical and chemical features. However, the repellency of MXene-based films is relatively poor to common organic dyes. In view of the excellent repellency of GO membranes, composite membranes (GO/MXene) that derived from GO and MXene have been explored recently. The combination of MXene and graphene can effectively combine their respective advantages, and the combined material has a large specific surface area and specific capacity. In Figure 2g, it can be seen that the space between adjacent nanocryphals in GO/MXene composite films forms nanoscale channels that allow water to pass through and repel pollutants. 

As shown in Figure 2h, the surface of the GO/MXene composite membrane also has obvious wrinkles. These morphological characteristics can be regarded as the inherent characteristics of the 2D ultra-thin layered nanomaterial stack. In addition, Ti elements are uniformly distributed on the surface and cross section of the GO/MXene composite film (mass ratio = 1/1), which further improves the uniformity of GO and MXene nanosheets in the composite film. Subsequently, Liu et al. [60] extensively evaluated the structure and physical and chemical properties of the GO/MXene composite film through XRD, Fourier-transform infrared spectroscopy (FT-IR), X-ray photoelectron spectroscopy (XPS) and contact angle analysis. They pointed out that the combination of graphene oxide layered structures provides more low-friction nanotube channels, thereby improving water permeability. The filtration performance of GO/MXene composite membranes was then evaluated from the water flux and the removal rate of organic pollutants in water, respectively. The water flux of the pure GO membrane is 6.5 Lm^−2^ h^−1^ bar^−1^, which increased with increasing the incorporation of MXene. The reason is that, on one hand, the presence of MXene reduces the π–π interaction between successive GO nanosheets and increases the interlayer spacing between nanosheets, thereby endowing higher water flux. On the other hand, by introducing MXene into the composite membrane, the interaction (hydrogen bond) between water molecules and oxygen-containing functional groups in the oxidation zone of the GO nanosheets is weakened, thereby reducing the water flow resistance and thus increasing the water flux. In the removal of organic pollutants, due to the molecular sieve (mainly) of the composite membrane and the electrostatic interaction between the layers, the composite membrane has excellent repellent performance. In the experiment, it was found that the removal efficiency of GO/MXene film for small molecule dyes was close to 100% (99.5% of NR, MB, CV, and BB) [60]. Moreover, the as-prepared GO/MXene membranes were verified to have excellent selectivity and permeability with satisfied stability in aqueous conditions presenting huge potential for their future development and practical application toward water purification.

### 3.2. 3D Structures

#### 3.2.1. Self-Assembled Structure

However, the stacked MXene sheets easily slip between them, showing poor strain resistance [64]. Fortunately, in recent years, researchers have used the high aspect ratio structure of MXene sheet and its highly functional surface to assemble into a multifunctional columnar structure through layer-by-layer (LbL) self-assembly technology. Self-assembly is considered as a promising strategy to transform various 2D structured materials into 3D materials. It can transform the layered structure into a macroscopic material with a hierarchical structure and novel function.

**Figure 3:**
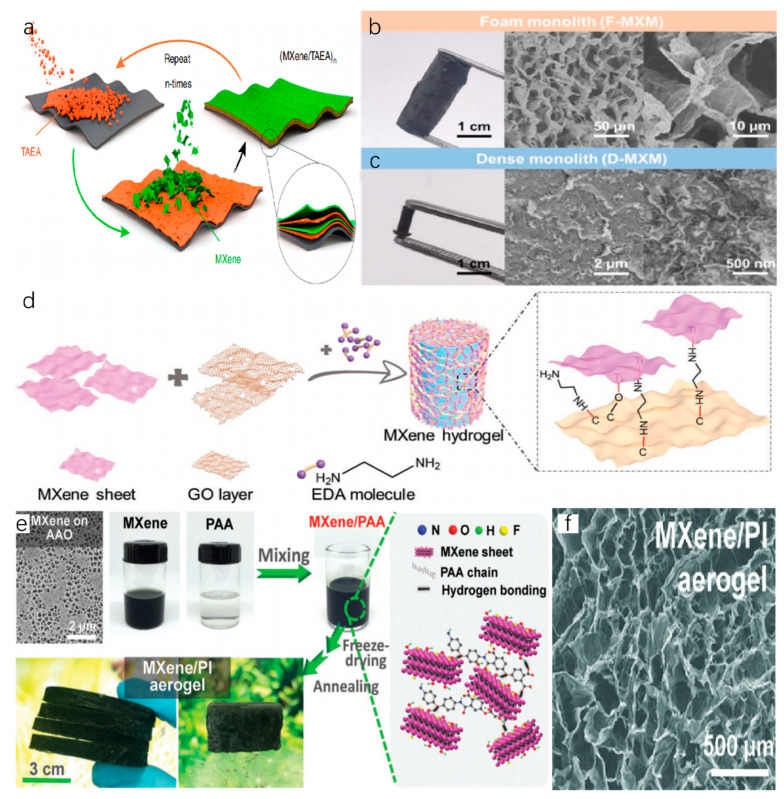
(**a**) Schematic illustration of the LbL self-assembly of (MXene/TAEA)_n_ multilayer films onto planar substratesa. Reproduced from [65], with permission from Nature Publishing Group, 2019. (**b**,**c**) The morphology and microstructure of MXM: freeze-drying (**b**) and capillary-drying (**c**). (**d**) MXene-based Hydrogel synthesis diagram. Reproduced from [66], with permission from Wiley, 2019. (**e**) A schematic illustrating the fabrication process and digital images showing the light weight of the MXene/PI aerogel, which can rest on the top of a dandelion. (**f**) The SEM image of MXene/PI aerogel, which has a compact interface and an interconnected porous structure. Reproduced from [67], with permission from Wiley, 2018.

LbL is usually a cyclic process in which two oppositely charged substances are alternately deposited on a substrate to form a multilayer structure with a thickness proportional to the number of layers (Figure 3a). In the LbL architecture, different layers are held together through electrostatic interactions, covalent bonds and hydrogen bonds or ionic charge transfer. Tian et al. [65] reported the fabrication of columnar 2D MXene multilayer by using LbL self-assembly, in which a small molecule of tris(2-aminoethyl) amine (TAEA) and Ti_3_C_2_T_x_ MXene have been applied to form multilayer colloidal dispersion (MXene/TAEA)_n_, where n represents a double Number of layers. 

In the process of studying the growth behavior of LbL MXene multilayer films, it was found that the thickness of (MXene/TAEA)_n_ increases linearly with the number of double layers n. The mass load of the multilayer structure also linearly increases with the increase of n. This linear behavior is a characteristic of successful LbL self-assembly, indicating a complete alternation of two different materials during the LbL process. The microstructure of the LbL film was analyzed by AFM, and it was found that the MXene monolayer film was stacked face to face. This indicates that there was no slippage between layers during the assembly process. In addition, the XRD pattern of the (MXene/TAEA)_n_ multilayer film shows an ordered structure similar to the original MXene film, which is due to the orderly stacking of the MXene film base. The small size of TAEA causes it to form sub-nanometer gaps between MXene flakes, resulting in close interface contact between flakes analogous to pure MXene film. During the assembly process, the average interlayer distance between the MXene flakes in the multilayer film will increase. This is due to the pillar effect produced by the complex interface interaction between the MXene flakes and TAEA. Subsequently, the contact angle measurement showed that the TAEA layer was more hydrophilic comparing to the MXene layer. Therefore, the TAEA column in the columnar architecture increased the availability of the aqueous electrolyte to the MXene intermediate layer to achieve effective ion transfer and diffusion. Thus, highly conductive pillared MXene multilayers and probably other 2D heterostructures can be fabricated at large-scale through this strategy. 

#### 3.2.2. Hydrogels

The hydrogel with similar advantages of biological tissues has triggered a research boom for more than ten years. In recent years, a series of functional hydrogels with high mechanical properties, antibacterial properties, filtration properties and electrical conductivity have been prepared through the precise design of structural composition, synthesis methods and fillers. These functionalized hydrogels have wide applications in the fields of biomedicine and environmental protection [68]. For example, 3D hydrogels based on the new 2D material MXene and having a dense microstructure have been developed. When used as an electrode, the 3D hydrogel with porous structure can increase the electron transfer rate, thereby obtaining excellent weight capacitance and rate performance. Moreover, in different drying processes, MXene hydrogels are transformed into MXene monomers (MXMs) with completely different morphology and microstructure. As shown in Figure 3b and c, it can be found that the formed aerogel had a loosely organized porous foam structure (F-MXM) or a dry gel with a dense structure (D-MXM).

A 3D MXene hydrogel (MXH) formed by a self-assembly method in the presence of GO and ethylenediamine (EDA) has been reported. Figure 3d presents the schematic diagram of the formation process of MXene hydrogel. EDA offers the crosslinking sites for the MXene flakes and the partially reduced GO sheets. The GO sheets with intrinsic flexibility plays a key role on neutralizing the rigidity of the MXene flakes and constructing the 3D macrostructures. Then the assembly of MXene flakes are driven by the van der Waals force between the MXene flakes and assisted by the π–π interaction of GO sheets crosslinked with MXene. In MXH, Figure 3b shows SEM image of the freeze-dried sample (F-MXM). It can be seen from Figure 3b that the constructed MXH has a well-defined and interconnected 3D porous network. The pore size of the network ranges from submicron to several microns, and the pore walls are composed of stacked MXene flakes, which are cross-linked by GO flakes. When applying with capillary drying for eliminating water from the hydrogel, a very hard material can be obtained with significant volume shrinkage and a dense microstructure (D-MXM) (Figure 3c). D-MXM has a high specific surface area of 196 m^2^ g^−1^ far exceeding the specific surface area of previously reported MXene-based materials. F-MXM and D-MXm have different applications due to their different structure. Freeze-dried porous 3D MXene foam hydrogels (F-MXM) shows high adsorption performance in removing various organic liquids and heavy metal ions. In addition, the dense 3D MXene monolith hydrogels(D-MXM) has high Young’s modulus, volume, and hardness, and can be used in clean environments, machinery and energy storage [66].

#### 3.2.3. Aerogels

Assembling the MXene sheet into a macroscopic 3D structure is an effective means to utilize its extraordinary performance [69]. However, due to the weak interaction between MXene sheets, it is difficult to build an independent, mechanically flexible, 3D MXene sheet skeleton. Another point is that while assembling the 2D MXene sheet into a 3D macro structure, it is necessary to overcome the serious re-stacking problem of the 2D MC MXene sheet.

Recently, researchers have proposed an interface enhancement strategy to build a multifunctional, superelastic and lightweight 3D MC MXene structures by connecting a single MC MXene sheet with polyimide macromolecules. Figure 3e shows the manufacturing process of MXene/PI aerogel. By etching and exfoliating the Ti_3_AlC_2_ MAX phase precursor with LiF/HCl solution for selectively removing the Al layers, high-quality MXene sheets are obtained. The MXene suspension was mixed with the poly (amic acid) (PAA) solution, and the resulting mixture was then frozen and lyophilized. The formed MXene/PAA aerogel was thermally annealed in an Ar atmosphere at 300 °C to induce PAA to polymerize to form PI macromolecules. The synergy between MXene and PI also makes the aerogel have good flexibility and mechanical stability. MXene/PI aerogel is light in weight and can be placed on dandelion (Figure 3e). In addition, MXene/PI lightweight aerogel has greater reversible compressibility, outstanding fatigue resistance and strong electrical conductivity. Its excellent mechanical flexibility and conductivity make aerogels have broad application prospects in damping, microwave absorbing coatings and flexible strain sensors.

In order to study the 3D structure of MC MXene-based aerogels, Liu et al. [67] compared the microstructure of Mxene aerogel, PI aerogel and Mxene/PI aerogel. Pure MC MXene aerogel presents a loose and disordered porous structure, with sheet-like structures partially overlapping and weakly connected to each other. This makes the fragile structure easy to collapse neatly under slight pressure. As for the neat PI aerogel, its PAA aerogel precursor undergoes severe shrinkage during thermal annealing, resulting in poor elasticity and high density. Although it has a porous network with smooth pore walls, it cannot be used in practical applications. Interestingly, by simply integrating MC MXenes sheet and PI, the manufactured MXene/PI aerogel products have compact interfaces and interconnected porous structures (Figure 3f). The MC MXene sheet is tightly encapsulated by the PI layer, and the framework of the sandwich structure is formed through the effective interface of the PI macromolecular chain (Figure 3f). The polar interface interaction between the surface ends of MC MXenes and the polar PI chains helps to form a strong network. The MC MXene sheets are tightly connected and constrained by the PI chain, forming a strong porous frame as a rigid skeleton. These skeletons are staggered and connected to form a uniform and complete 3D network aerogel on a macroscopic scale. This structure is conducive to load transfer, rapid transmission of electrons, and has great resistance to cyclic strain and deformation.

## 4. Applications of the MC MXene-Based Nanomaterials

MC MXene has the characteristics of high specific surface area and high conductivity, and also has the advantages of flexible and adjustable components, and controllable minimum nanometer layer thickness. It has shown great potential in the fields of energy storage, adsorption, photocatalytic, heavy metal adsorption etc. In this section, we will mainly introduce the applications of 2D MC MXenes in these directions. To the best of the authors knowledge, most majority of the reported applications of existing MC nanomaterials are based on Ti_3_C_2_ and their complexes, hence in this section, we primarily focus on Ti_3_C_2_ and Ti_3_C_2_ composites while discuss other MXene-based nanomaterials briefly.

### 4.1. Chemical Batteries

With the emergence of miniature wearable/portable electronic products, most of micro-electromechanical systems (MEMS), epidermal and implantable medical and wireless sensor networks need to develop electronic energy storage devices that match their functions. Therefore, electrochemical energy storage devices have recently received more and more attention. In this part, we will focus on the latest application of MC MXene in energy storage devices.

**Figure 4:**
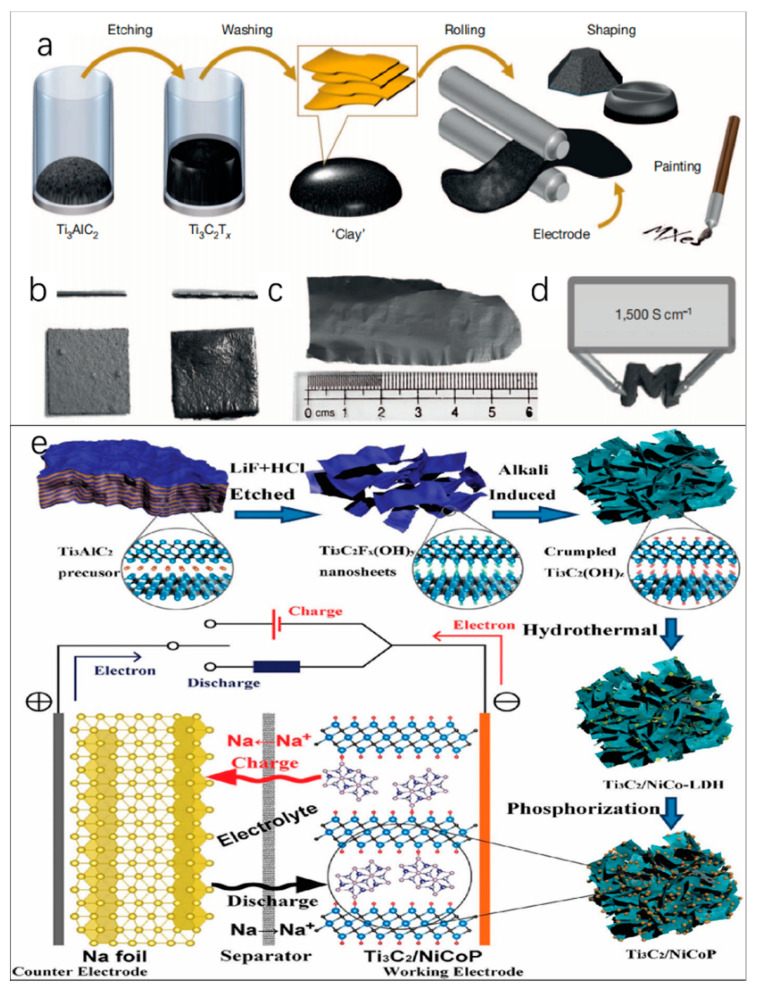
Schematic of MXene clay synthesis and electrode preparation: (**a**) the schematic of MXene clay synthesis and electrode preparation processes. (**b**) When the dried sample (left) is hydrated (right), it will expand. (**c**) The produced flexible, free-standing film in a roller mill between water-permeable membranes. (**d**) Mold “clay” into the letter M and dry it to obtain a conductive solid. Reproduced from [42], with permission from Nature Publishing Group, 2014. (**e**) The intercalation of ions in layered compounds has been used for a long time in energy storage devices such as batteries and electrochemical capacitors. Reproduced from [72], with permission from Royal Society of Chemistry, 2019.

Various renewable energy storage technologies have been developed, such as lithium-ion batteries, sodium-ion batteries, lithium-sulfur batteries, and supercapacitors, but there are certain shortcomings in traditional manufacturing technologies. For example, lithium-ion batteries cannot quickly release energy at low power, the performance of sodium ion batteries decays faster and, most super capacitors are costly [70], etc. Therefore, it is of practical significance to develop novel energy storage equipment. As a typical representative of the emerging 2D layered transition metal carbide, Ti_3_C_2_T_x_ has the advantages of metal conductivity, plastic layer structure, small band gap, and functionalized surface hydrophilicity. The application of such the 2D material in the field of energy storage has been extensively studied [71]. In the earliest times, Ghidiu et el. [42] reported the fabrication of a 2D titanium carbide ‘clay’ through etching TiALC with LIF and HCL and used as the electrode material for energy storage. Figure 4a shows a schematic diagram of the synthesis of MC MXene clay and the fabrication of electrode process. The MAX phase is etched in an acid and fluoride salt solution, and then washed with water to remove the reaction products and to raise the pH to neutral, thereby forming a clay-like deposit. The clay can be rolled into a flexible film to produce a conductive object of the desired shape or diluted to prepare a conductive coating. After hydration, the volume of the resulting hydrophilic material expands, and it can be shaped like clay and dried into a highly conductive solid, or rolled into micrometers thick films (Figure 4b–d). Additive-free films of this titanium carbide clay have volumetric capacitances of up to 900 F/cm^3^, with excellent cyclability and rate performances. It was found that the as-synthesized conductive 2D Ti_3_C_2_T_x_ “clay” with high volumetric capacitance exhibited huge application potential in electrochemical energy storage. So far, more and more research on MC MXene in energy storage have been gradually reported.

In recent years, studies have shown that Ti_3_C_2_T_x_ MXene film has the characteristics of easy insertion and effective ion diffusion, so it has attractive performance. For example, people used HFA to etch aluminum selectively to obtain Ti_3_C_2_T_x_ for using as electrode materials. It has been found that when the battery was charged and discharged, there was enough space for lithium ions to be quickly inserted into the layer of the battery without causing structural damage. The obtained result showed that Ti_3_C_2_T_x_ MXene has satisfactory activity as an electrochemical material. Recently, in order to verify the stability of the MXene material, electrochemical behavior of the material on the supercapacitor was further tested. For instance, Syamsai et al. [73] used a simple ball milling method to prepare titanium carbide Ti_3_C_2_T_x_ from TiO_2_, aluminum, and graphite powder. The exfoliated layered structures facilitate the penetration of electrolyte into the electrode material, thereby giving a path for ions to penetrate into the electrode. Electrochemical investigation have shown that in a 6M KOH electrolyte and at a scan rate of 1 mV/s, the maximum specific capacitance of Ti_3_C_2_ and Ti_2_C is about 447 F/g and t 248 F/g, respectively. These excellent properties indicate that the electrode materials can be used for supercapacitor applications. In general, this work provides safe, effective, and detailed investigation of titanium carbide as chemical material for chemical batteries. 

Although MXene exhibits excellent electrochemical properties, it is still an ongoing issue due to the restacking of the ultrathin Ti_3_C_2_T_x_ layers during repeated insertion/extraction processes. In order to overcome this shortcoming, some researchers have designed a unique 3D porous material based on MXene to avoid the re-stacking of 2D nanosheets. For example, Zhao et al. [72] developed a new strategy to connect NiCoP bimetallic phosphide nanoparticles with alkali-induced 3D interconnected wrinkled porous Ti_3_C_2_ MXene as an anode for high-efficiency sodium-ion batteries. Figure 4e illustrates the synthesis process of Ti_3_C_2_/NiCoP hybrid and the mechanism of the half-cell. The interconnected 3D Ti_3_C_2_ wrinkle structure established a 3D conductive network with rich openings and huge surface area, thereby providing 3D conductive and unobstructed channels for a rapid charge transfer process and electrolyte storage, and the electrodes are in full close contact with the electrolyte. The synergistic effect of NiCoP and Ti_3_C_2_ improved the electrochemical performance of synthesized materials, providing a new method for constructing electrodes of high-performance energy storage devices. 

Although there are already more than 20 other members in this large-scale 2D MC material family, research on MC MXene used in supercapacitor applications is mainly focused on Ti_3_C_2_. Currently, other MC MXene research is also increasing, such as Ti_2_C, Mo_2_C, V_2_C and Mo_1.33_C, which have achieved promising applications in aqueous electrolytes. Recently, Shan et al. [74] reported the electrochemical behavior of vanadium carbide MXene, V_2_C, in three water electrolytes. Excellent capacitance values were obtained in all three solutions. The maximum specific capacitance values obtained in 1 M H_2_SO_4_, 1 M KOH and 1 M MgSO_4_ are 487 F/g, 184 F/g and 225 F/g, respectively, outperforming current commonly used similar electrode capacitance values of several microns thick. It should be noted that the best performance can be obtained in 1 M H_2_SO_4_, and the capacitance can reach 487 F/g at 2 mV/s, which has far exceeded the capacitance value of Ti_3_C_2_, Mo_1.33_C and Mo_2_C MXene. The encouraging results suggest the potential to further explore vanadium carbide crystals for energy storage applications. In another case, Mei et al. [75] prepared Mo_2_C MXene successfully without fluorine end by UV-induced selective corrosion of Mo_2_Ga_2_C precursor. When synthetic Mo_2_C MXene is used as the anode material of rechargeable batteries, both lithium-ion batteries and sodium ion batteries have obtained excellent rate capability and cycle stability. What is important is that the assembled flexible Mo_2_C MXene battery also exhibits excellent capacity retention capability under bending. This flexibility also gives Mo_2_C MXene great potential for shaping electrode shapes in energy storage applications.

### 4.2. Supercapacitors

Supercapacitors refer to a new type of energy storage device between traditional capacitors and rechargeable batteries. It not only has the characteristics of fast charging and discharging of capacitors, but also has the energy storage characteristics of batteries. According to the working principle, the supercapacitor can be divided into two forms. One of them is biased towards physical effects, in which capacitors store charge through an electrostatic field. In other words, their positive and negative charges are stored by polarized electrodes. This type of super capacitors are called electric double layer capacitors (EDLC) [76]. The other is dominated by chemical effects, and the high capacity mainly depends on the electroactive material. The electroactive material undergoes chemical adsorption/desorption or oxidation-reduction reaction on the electrode surface by applying a voltage. This type is called a pseudo capacitor.

MC MXene is an excellent electrode material for supercapacitors. Because of the abundant functional groups on the surface of MC MXene, in the water electrolyte, metal ions can combine with water molecules to form an electric double layer structure on the surface of MC MXene. In addition, redox reactions may occur on the surface of MC MXene, which will improve the capacitive performance of MC MXene-based supercapacitors. Similar to other 2D materials, MC MXenes can be readily manufactured by the vacuum filtration. Nevertheless, MC MXene nanosheets are liable to re-stacking during the self-assembly process, leading to poor electrolyte penetration. To fully utilize the electrochemical energy storage capacity of MC MXenes, Zhang et al. [77] synthesized macroporous MC MXene membranes as electrodes for supercapacitors through ion-assisted self-assembly. The ions produced by the hydrolysis of the chloride salt can be adsorbed on the surface of the nanosheets, thereby reducing the negative surface charge of the MXene nanosheets, and causing the nanosheets to aggregate and form a 3D macroporous structure under the action of van der Waals forces. The electrodes assembled by K^+^ and H^+^ showed exciting results, that is, the area capacitance is 1025 mF cm^−^^2^ (427 Fg^−1^) and 935 mFcm^−^^2^ (374 Fg^−1^) and has excellent rate performance, its excellent capacitance is higher than the re-stacked MXene (768 mF cm^−^^2^). The results showed that the ion-assisted self-assembly of macroporous MXene films has broad application prospects in supercapacitors. Moreover, the method is expected to be applied to lithium-ion batteries, water electrolysis, water pollution treatment, seawater desalination and other fields.

**Figure 5:**
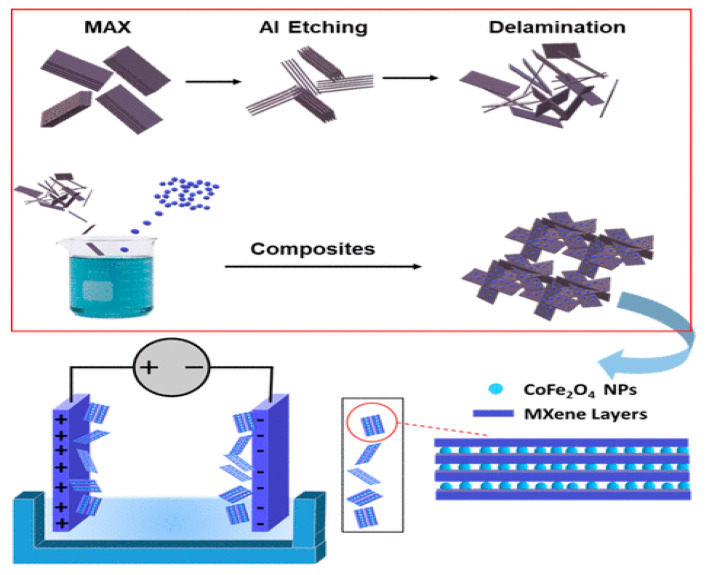
Schematic illustration showing the synthesis of CoF NP-decorated MXene and its application as a supercapacitor electrode. Reproduced from [76], with permission from American Chemical Society, 2020.

In order to overcome the obstacle of the supercapacitance performance of MC MXene as an electrode material by the layer pressure caused by the interaction of functional groups, a method of adding spacers between the MXene layers can also be used. For example, Ayman et al. [76] explored the application of MXene and its composite materials with cobalt ferrite nanoparticles (CoF NPs) in hybrid supercapacitors similar to batteries. Use nanoparticles as intermediate spacers between MXene layers. The introduction of CoF NPs between MXene layers to avoid overlap of MXene. MXene flakes are synthesized by chemical etching (Ti_3_AlC_2_) MAX phase, and then layered by ultrasonic. Delamination of MXene flakes can provide a large surface area, and the surface area can be further increased by adding nano-CoF between the layers, as shown in Figure 5. Through electrochemical research, it is proved that the electrochemical performance of the composite material (CoF/MXene) is better than that of a single ferrite or MXene. Taking into account the characteristics of MXene and CoF, introducing CoF into MXene can better improve the total capacitance and stability of MXene. CoF/MXene composite not only combines the characteristics of ferrite and MXene, but also acts as a hybrid electrode to improve the energy storage capacity of battery-type super capacitors. In composite materials, CoF NPs maintain the separation of the MXene layer, thereby promoting the transport of electrons and ions. Experimental data shows that the maximum specific capacitance of CoF NPs, MXene, and CoF/MXene composites was observed to be about 594, 1046.25, and 1268.75 Fg^−1^ at 1 A g^−1^, respectively. These comparative studies confirm that the hybrid composite electrode has a good capacitance value. In addition, the CoF/MXene composite also has good cycle stability, with a capacitance retention rate of 97% until 5000 cycles. The synergistic effect of CoF NPs and MXene can help researchers further explore efficient energy storage devices for MXene/NPs composite materials.

### 4.3. Water Splitting

Due to rapid growth of industry and population, coal, oil, natural gas and other fossil fuels have been consumed in large quantities, causing energy crisis and environmental problems [45]. The development of renewable and efficient fuel resources (e.g., hydrogen energy) to replace traditional unsustainable fossil fuels has received tremendous attention. At present, photocatalyst has been widely used in environmental purification, advanced new energy, and other fields. However, the exploitation of cheap and efficient photocatalytic emission reduction materials is still a challenge [78]. The principle of photocatalysis is that under the excitation of light, electrons transition from the valence band to the conduction band. In this way, photo-generated electrons are formed in the conduction band and photo-generated holes are formed in the valence band. Using photo-generated electron-hole pairs to redox performance can degrade organic pollutants in the surrounding environment and produce hydrogen and oxygen by photohydrolysis. 

**Figure 6:**
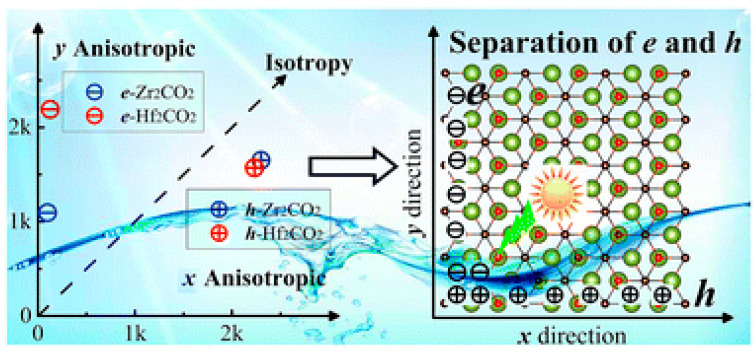
Schematic diagram of the relationship between carrier mobility and direction of 2D Zr_2_CO_2_ and Hf_2_CO_2_ and the separation of photogenerated electron-hole pairs. Reproduced from [81], with permission from Royal Society of Chemistry, 2016.

A suitable photocatalyst generally needs to meet two requirements. The first is that it must have a suitable conduction band and valence band position, and the second is that it has effective electron-hole separation ability and excellent visible light response characteristics. In pollutant applications, the valence band potential must have sufficient oxidation performance. In photohydrolysis applications, the potential must meet the requirements for generating hydrogen and oxygen. In recent years, 2D transition metal material MXene can fully meet the above conditions with excellent photocatalyst characteristics [79]. Due to its flexible and adjustable element composition, regularity of the layered structure and excellent electrical conductivity, MXene has been used as a suitable substitute for promoting photocatalytic performance [80]. The important reason is that the band gaps of MXenes materials are all greater than 1.23 eV, which is the minimum requirement for photocatalytic water splitting. For example, the 2D Zr_2_CO_2_ and Hf_2_CO_2_ of the MXenes family are promising photo-splitting water catalysts, which can effectively separate photoelectrons, and have high carrier mobility. Guo and his colleagues [81] systematically studied the photocatalytic performance of 2D MXene as a photocatalyst based on density functional theory and deformation potential energy theory. In the electronic structure of MXenes, the energy gap and band edge positions of 2D Zr_2_CO_2_ and Hf_2_CO_2_ meet the requirements of photocatalytic water splitting. When 2D Zr_2_CO_2_ and Hf_2_CO_2_ MXenes are used for photocatalytic water separation, photoelectrons and holes will migrate in the direction shown in Figure 6, thereby realizing the effective separation of photoelectrons and holes. At the same time, 2D Zr_2_CO_2_ and Hf_2_CO_2_ also show very good light absorption at visible and ultraviolet wavelengths. In addition, both Zr_2_CO_2_ and Hf_2_CO_2_ have unexpectedly large anisotropic carrier mobility, which means that electrons have an absolutely dominant tendency to migrate in the vertical direction, while holes tend to diffuse in the horizontal direction [81]. It can be concluded that a large directional anisotropic carrier mobility promotes the migration and separation of photogenerated electron-hole pairs, thereby improving the efficiency of photocatalytic water splitting. This example focused on the application of MXene as a photocatalyst, and further guided the design of novel MXene in photocatalysis.

Recently, significant progress in the conversion of 2D MXene-based photocatalysts into solar fuels, reasonable design and specific effects of MXene photocatalysts for the production of photocatalytic solar fuels have been reported [82]. For example, Xie et al. [83] introduced various roles of MXene in preparing composite photocatalysts and improving their activity and stability, clarified their application prospects in photocatalysis, and provided beneficial basis for the rational design and synthesis of efficient and stable MXene-based photocatalysts. It was found that the performance of the layered nanostructure MXene in environmental and energy photocatalysis is amazing, especially those composite materials hybridized with MXene fully demonstrating the excellent characteristics of this special structure [83]. At present, MXene composite materials have been applied to the research of dye-sensitized photocatalysis. Sun et al. [84] oxidized Ti_3_C_2_ MXene in water at 60 °C for different times to form TiO_2_/Ti_3_C_2_ on amorphous carbon (AC) composites. The oxidized MXene was adopted as the photocatalyst in a dye sensitization system for hydrogen evolution to replace noble metal promoters such as Pt, which offers an opportunity of for 2D MXenes in dye-sensitized photocatalytic hydrogen.

### 4.4. Photocatalysts for Degradation of Pollutants

**Figure 7:**
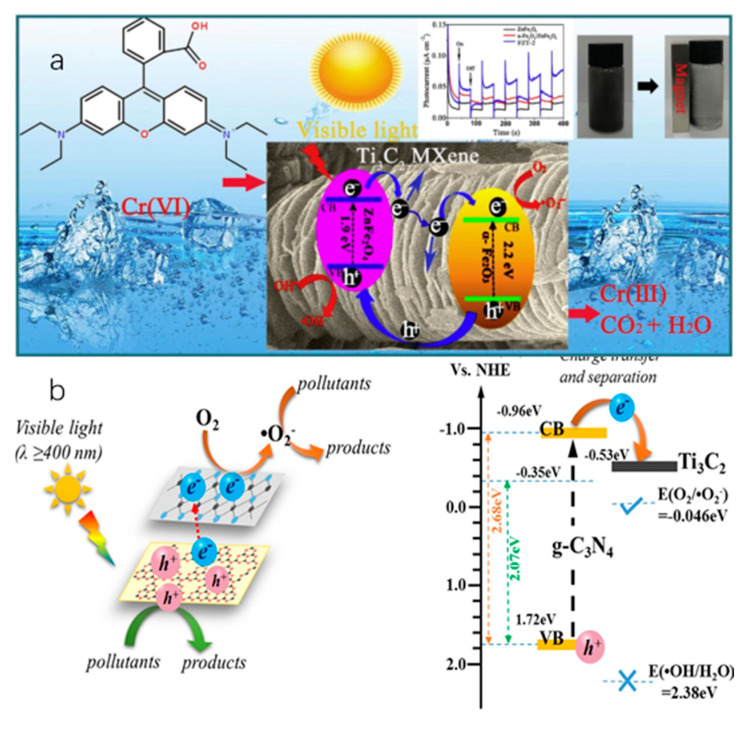
Degradation mechanism of pollutants by photocatalyst prepared with MXene: (**a**) Schematic mechanism of magnetic α-Fe_2_O_3_/ZnFe_2_O_4_@Ti_3_C_2_ MXene photocatalyst. Reproduced from [85], with permission from Elsevier, 2020. (**b**) Schematic diagram for the decomposition of organic pollutants under visible light irradiation. Reproduced from [87], with permission from Elsevier, 2019.

As a photocatalyst, MXene can not only generate new energy, but also shows amazing potential in the environmental governance of purifying organic pollutants. Zhang et al. [85] used a one-step hydrothermal synthesis method to prepare a magnetic α-Fe_2_O_3_/ZnFe_2_O_4_ heterojunction. The magnetic α-Fe_2_O_3_/ZnFe_2_O_4_ heterojunction is dispersed on the surface of Ti_3_C_2_ MXene by the ultrasonic-assisted self-assembly method, and α-Fe_2_O_3_/ZnFe_2_O_4_@Ti_3_C_2_ MXene photocatalyst can be easily obtained. And found that α-Fe_2_O_3_/ZnFe_2_O_4_@Ti_3_C_2_ MXene had a high photocatalytic ability, which eliminated rhodamine B (RhB) pollutants and toxic Cr (VI) in water. The excellent photocatalytic ability was attributed to numerous heterostructure interfaces, increased visible light collection and high electrical conductivity. In addition, the unique nanostructure of α-Fe_2_O_3_/ZnFe_2_O_4_@Ti_3_C_2_ MXene accelerated the separation and transfer of photogenerated electrons, thereby significantly improving its photocatalytic performance (Figure 7a). More importantly, the α-Fe_2_O_3_/ZnFe_2_O_4_@Ti_3_C_2_ MXene photocatalyst was reusable, which improved the material utilization rate. For now, with the research on 2D carbonized materials, MXene has also achieved the degradation of antibiotic drugs accumulated in water all the year round. Antibiotics are the most frequently used drugs in modern times, but such the drug molecules cannot be completely absorbed by the human body [86], which leads to a vast amount of drugs to be excreted through feces and urine. These drugs accumulate in natural water, causing severe water pollution. Fortunately, the photocatalytic decomposition of the antibiotic ciprofloxacin (CIP) by g-C_3_N_4_/Ti_3_C_2_ composite (CNTC) has been reported by Su and co-workers [87]. Their study on the photocatalytic performance mechanism of CNTC to CIP showed that the degradation rate was greatly increased after adding Ti_3_C_2_ to form a heterostructure. The photocatalytic degradation mechanism isillustrated in Figure 7b. Under visible light irradiation, the generated electrons (e^−^) can be quickly transferred from g-C_3_N_4_ to Ti_3_C_2_, and the abundant photo-generated holes (h^+^) on g-C_3_N_4_ can effectively degrade CIP. Meanwhile, the dissolved oxygen in the solution can be reduced to the superoxide compound O_2_ by the electrons obtained on the surface of Ti_3_C_2_, which can attack CIP molecules, thereby increasing the degradation rate of CIP. Further studies have shown that the TiO_2_/Ti_3_C_2_T_x_ composite material synthesized by hydrothermal method could also be used for the photodegradation of the antiepileptic drug carbamazepine (CBZ) [87].

### 4.5. Heavy Metal Adsorption

Heavy metal pollution has always been one of the most serious environmental issues in the ecological environment in the world, especially these heavy metal ions with high cytotoxicity [88]. In addition, heavy metal pollution has significant characteristics such as non-biodegradability, high cytotoxicity to organisms and threat to human living environment [88]. In recent years, great attentions have been paid to the treatment of heavy metal pollution, and various effective methods, including coagulation, electrochemistry, ultrafiltration, chemical oxidation, catalytic reduction, dialysis, and ion exchange have been developed [89]. However, these methods suffer from disadvantages including high energy consumption, high cost and secondary pollution [90]. The difference between the adsorption and the above method is that it is simple to operate and has little impact on the environment. As far as the adsorption method is concerned, it is of key importance to select and prepare a promising and suitable adsorbent with the advantages of low cost, good chemical stability, good selectivity and easy preparation [91].

MXene possess a large specific surface area, hydrophilicity and abundant highly active surface sites [92,93], which has been proven to adsorb and remove heavy metal ions, organic dyes, radionuclides, gas molecules and other environmental pollutants [91]. More importantly, MXene materials have abundant surface functional groups, which can provide more adsorption sites for heavy metal ions, and have been widely used in the field of heavy metal adsorption [94]. Recently, it was found that the 2D layered MXene material can be alkalization intercalated to achieve heavy-metal ion adsorption [95]. For example, Guo et al. [95] used first-principles and DFT to explain the adsorption kinetics of heavy metal ions and the influence of intercalation sites on adsorption. When the coverage of heavy metal ions is greater than 1/9 of a single layer, the 2D alkalized intercalated MXene (alk-MXene) Ti_3_C_2_(OH)_2_ exhibits strong heavy metal ion absorption. The calculation results showed that 2D alk-MXene is an effective adsorbent for removing heavy metal ions in water. The advantage of 2D alk-MXene to adsorb heavy metal particles is that the formation of the hydrogen potential trap provides a hydrogen potential composed of hydrogen atoms of Ti_3_C_2_(OH)_2_ around heavy metal and O atoms below the heavy metal atom. Thus, the reverse spin electron pairs are sufficient to capture heavy metal atoms [96]. 

**Figure 8:**
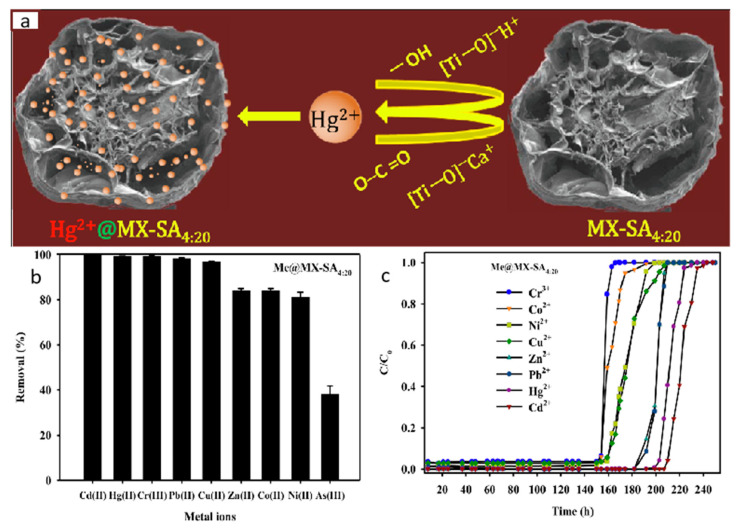
Adsorption performance of MX-SA_4:20_ in a multi-component system: (**a**) Diagram showing that Hg^2 +^ ions are absorbed by MX-SA_4:20_ spheres. (**b**) The batch system simultaneously adsorbs eight toxic metal ions. (**c**) Breakthrough curve in fixed adsorption of multi-ions. Reproduced from [97], with permission from Elsevier, 2019.

In recent years, MXene can be hybridized with other materials to fully realize its performance in the field of heavy metal adsorption. For example, Shahzada et al. [97] adopted different concentrations of titanium sulfate MXene and sodium alginate (SA) to prepare a 2D titanium carbide core (Ti_3_C_2_T_x_) shell aerogel ball (MX-SA) for mercury ion removal. The spherical porous structure facilitates the capture of metal ions during the adsorption process. The hydroxyl and carboxyl groups in alginate are well-known heavy metal binding groups. The MX-SA_4:20_ sphere has a special adsorption capacity of 932.84 mg/g for Hg^2+^, which is one of the highest adsorption capacities among adsorbents reported so far. The microsphere structure with unique internal structure, high porosity and large specific surface area makes the prepared MX-SA have excellent adsorption performance and can remove heavy metals in water. The adsorption mechanism is shown in Figure 8a. The binding group [Ti-O] ^−^H^+^ and Hg^2+^ on the inner surface of the ball exhibit strong metal-ligand interaction. In addition, the formation of [Ti-O] ^−^Ca^+^ groups during the synthesis process was conducive to enhance Hg^2+^ uptake. The adsorbent has high single-component and multi-component removal efficiency, in which the efficiency of Hg^2 +^ is 100%, and the efficiencies of five heavy metal ions are greater than 90%, as shown Figure 8b,c. This research also shows that the spherical manufacturing process using 2D MXene can be expanded and scaled, enabling large-scale synthesis of multi-pollutant remediation at the industrial level.

At present, it is extremely challenging to repair the environmental damage caused by heavy metals, especially radioactive heavy metals [98]. For example, thorium and uranium are both radioactive and toxic heavy metal, and are extremely harmful to the environment. Radioactive substances invade the human body and can cause chronic diseases, which are mainly manifested by weakened general functional status and significantly reduced resistance to infectious diseases. Therefore, studying the adsorption and separation of radioactive heavy metals is of great significance to environmental protection and human health. Due to the excellent hydrophilicity and abundant surface functional groups of 2D MXene material, researchers explored the performance of 2D Ti_2_CT_x_ MXene material to remove radioactive heavy metals Th(IV) [93]. Under different storage conditions, the adsorption capacity of Ti_2_CT_x_ in the hydrated state (Ti_2_CT_x_-hydrated state) showed greater enhancement than the dry state, which was mainly due to the larger interlayer space of Ti_2_CT_x_, and the easier entry of Th(IV) ions [96]. The maximum adsorption capacity of hydrated Ti_2_CT_x_ reached 213.2 mg/g, outperforming most common inorganic adsorbents. In addition, Ti_2_CT_x_-hydration had excellent adsorption selectivity for Th(IV) in the presence of metal ions. Using SEM combined with energy dispersive spectroscopy, XRD, and XPS analysis, Li et al. [98] characterized the morphology and structure of Ti_2_CT_x_ before and after adsorption, and proposed that the mechanism of Th(IV) removal is related to intra-sphere complexation. Their study proved that Ti_2_CT_x_ hydration is a promising method for thorium preconcentration and separation, which is of great significance to the environment protection [91].

In addition to the radioactive metal Th, the adsorption and separation of uranium have also been investigated. Zhang et al. [98] studied the adsorption behavior of uranium on V_2_C nanosheets, and found that all the studied uranyl species can stably bind hydroxylated MXene with a binding energy ranging from −3.3 to −4.6 eV. The binding process can be explained as strong adsorption by forming two U-O bonds with hydroxylated xylene. Besides, the axial oxygen atoms of uranyl ions formed hydrogen bonds with hydroxylated V_2_C, which further enhanced the adsorption. It was also found that the U-F bond was weaker than the U-O bond at the adsorption site, which indicated that the Mexne containing F are not very beneficial to the adsorption application of uranyl. This study expanded the application range of MXene nanosheets, making it not only to titanium carbide, but also of great application potential in environmental remediation.

### 4.6. Electromagnetic Shielding or Radiations

The vigorous development of electronic equipment and wireless communication improves people’s living standards, but produces severe EM interference and radiation, which greatly reduces data accuracy and communication quality, and even threatens human health. In order to attenuate EM pollution, conductive metal is traditionally used as a shielding material. But due to its corrosiveness, heavy weight, and inflexibility, it runs counter to the requirements of next-generation wearable smart protective equipment. So far, it is still a challenge to realize flexible, lightweight and robust electromagnetic interference (EMI) shielding candidates to replace metal materials [99]. At present, people can make full use of the interaction between incident EM radiation and shielding materials to effectively reduce EM radiation. When the EM wave hits the surface of the shielding material, a part of the EM wave is immediately reflected. The remaining waves are absorbed and dissipated as heat in the shield. The attenuation of EM radiation is mainly achieved through reflection, absorption and multiple reflection mechanisms [100]. Recently, as a shining star of 2D materials, MXene perfectly demonstrates its layered structure, excellent electrical conductivity, adjustable active surface, and outstanding mechanical strength. All these characteristics make it particularly attractive in a variety of applications, especially for the growing microwave absorption market and EMI shielding community [101]. 

Yun et al. systematically studied the EMI shielding performance of the 2D Ti_3_C_2_T_x_ MXene assembly film in the range of single-layer to multi-layer film thickness. A single-layer assembly film can shield 20% of EM waves, and a 24-layer film with a thickness of 55 nm can shield 99% of EM waves. This showed that the 2D Ti_3_C_2_T_x_ MXene assembly film has excellent EMI shielding effects. The main reason is the good electronic coupling between layers and excellent conductivity, which can effectively attenuate EM waves entering the material [101]. However, pure MXene has low mechanical properties. In order to meet the requirements of modern EMI shielding equipment, composite materials containing MXene have become a popular choice for EMI shielding materials due to their better mechanical properties and high processing performance. Some researchers have introduced highly conductive MXene into GO to effectively improve the conductivity of the obtained composite materials, but at the same time it will not increase the density significantly, thus obtaining a satisfactory EMI shielding performance. For example, Fan et al. [102] introduced highly conductive 2D Ti_3_C_2_T_x_ MXene nanosheets into GO, and obtained lightweight composite foams through freeze-drying and heat treatment. The introduction of MXene greatly improves the conductivity of GO, while maintaining the low density of the mixed foam, and has satisfactory EMI shielding performance.

2D Ti_3_C_2_T_x_ MXene composite materials have presented high potential in the field of EMI shielding. The main reason is that the EMI shielding mechanism of the 2D Ti_3_C_2_T_x_ MXene material has become more and more mature, so people can study various MXene composite materials on this basis to fully realize the EMI shielding performance of MXene. To date, the most commonly studied MXene Ti_3_C_2_T_x_, has been incorporated into different polymer matrices such as ultrahigh-molecular weight polyethylene (UMWPE), sodium alginate (SA) and polypyrrole (PPy). The MXene-polymer composites exhibited improved tensile strength, but good conductivity was still maintained at low polymer loadings [103]. SA is a linear polysaccharide copolymer extracted from seaweed. It is a potential ideal candidate for polymer matrix because of their abundant quantity, harmless to the environment, and strong mechanical properties. 

Recently, it has been found that adding SA to MXene materials does not destroy the conductivity of the material, which benefits to the application of composite MXene-SA in the field of EMI shielding. Shahzad et al. [103] reported the highly flexible MXene films (Ti_3_C_2_T_x_, Mo_2_Ti_3_C_2_T_x_) and nacre-like MXene-polymer composite films (Ti_3_C_2_T_x_-SA) with excellent EMI shielding performance. Ti_3_C_2_T_x_ flexible film has excellent electrical conductivity and EMI shielding ability. After adding SA to prepare polymer composite film, it presented good mechanical properties and stability, which can further expand practical applications. The mechanical flexibility and ease of coating provided by MXene and its composite materials enable them to shield surfaces of any shape while providing high EMI shielding efficiency. In practical applications, it can be used in the manufacture of shielding equipment to help eliminate the harm of electromagnetic radiation. This flexibility is far superior to existing EMI shielding materials and will pave the way for the application of 2D carbonized materials in the field of EMI shielding. 

**Figure 9:**
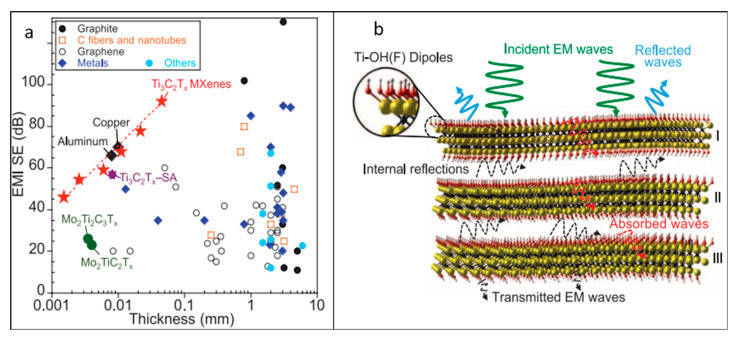
(**a**) EMI SE versus thickness of different materials. (**b**) EMI shielding mechanism. The incoming EM wave (green arrow) hits the surface of the MXene sheet. Due to the large number of charge carriers on the highly conductive surface, part of the electromagnetic wave was immediately reflected from the surface (light blue arrow), and the induced local dipole generated by the end base helps to absorb the incident wave through the MXene structure (blue dotted line). The transmitted wave changes when it encounters the next MXene sheet, causing multiple internal reflections (black dashed line) and more absorption. Reproduced from [103], with permission from AAAS, 2016.

Figure 9a shows the relationship between electromagnetic shielding efficiency (EMI SE) and different material thicknesses, in which the EMI SE of MXene film and Ti_3_C_2_T_x_-SA are among the best in the comparison table, outperforming the reported synthetic materials such as graphene, carbon nanotubes, iron oxides, ferrites, iron-aluminum-silicon alloys, and metallic-based filler polymer composites. Figure 9b illustrates the mechanism of EMI shielding. When EM waves hit the surface of the MXene sheet, due to the large amount of free electrons on the highly conductive MXene surface, some EM waves are immediately reflected. When the remaining waves pass through the MXene lattice structure, electrons interact with MXene to generate current, which leads to ohmic loss, and ultimately reduces the energy of EM waves. When the lower-energy transmitted wave encounters the next MXene sheet, it will go through the same process, causing multiple internal reflections (black dashed arrow in Figure 9b) and more absorption. Whenever an EM wave passes through the sheet, its intensity will be greatly reduced, thereby forming an EM wave that is attenuated or completely eliminated as a whole [103].

As mentioned above, the addition of Ti_3_C_2_T_x_ to a polymer matrix such as polyvinyl alcohol (PVA) is a common strategy to obtain the lightweight Ti_3_C_2_T_x_ film with high mechanical flexibility [31]. These insulating polymers are usually used as adhesives to penetrate into the MXene layer to enhance the mechanical properties of the synthesized MXene polymer composite. However, the conductivity will inevitably deteriorate if handled improperly, thereby affecting the shielding effect. In recent years, researchers have discovered a new additive material, namely the conductive material poly(3,4-ethylenedioxythiophene)-poly(styrene sulfonate) (PEDOT:PSS), and also studied the electromagnetic shielding performance of the ultra-thin Ti_3_C_2_T_x_/PEDOT: PSS hybrid film composed of (PEDOT: PSS) and MXene. Liu et al. [104] developed the ultrathin, flexible, and nacreous composite films with a “brick-like” structure using Ti_3_C_2_T_x_ MXene and conductive PEDOT:PSS through a vacuum assisted filtration process. The uneven penetration of Ti_3_C_2_T_x_ MXene and PEDOT:PSS significantly improves the mechanical properties of the composite membrane. In addition, the electromagnetic interference of different ratios of Ti_3_C_2_T_x_ and PEDOT:PSS composite films was also studied. The thickness of the ultra-thin polymer composite film with a weight ratio of Ti_3_C_2_T_x_ and PEDOT:PSS of 7:1 was only 11.1μm, but it showed a high EMI SE value of 42.10 dB. At the same time, compared with pure Ti_3_C_2_T_x_ MXene film, the tensile strength of the composite film was significantly increased from 5.62 MPa to 13.71 MPa, and the corresponding fracture strain increased from 0.18% to 0.29%. In addition, the hybrid film also has an excellent conductivity of 340.5 S/cm and an excellent specific EMI shielding efficiency of 19,497.8 dB cm^2^ g^−1^. Such the excellent electrical conductivity and EMI shielding efficiency promote the Ti_3_C_2_T_x_/PEDOT: PSS composite materials with great application potential as ultra-thin, lightweight and flexible EMI shielding materials to be used in smart wearable electronics and high-tech materials for military and national defense fields. For now, the performance of MXene/polymer membranes is closely related to the distribution of MXene flakes into composites. It has been reported that MXene flakes may aggregate during the manufacture of MXene/polymer membranes, resulting in multi-scale phase separation. Therefore, more efforts are needed to improve the dispersion of MXene flakes in the polymer matrix. This can be achieved by enhancing the interaction between the MXene flakes and the polymer matrix through proper polymer chain functionalization, thereby increasing the stability and life of the MXene/polymer membrane.

To sum up, special emphasis is given on various electronic devices employing MC MXene-materials with their distinct functions and performances, as listed in Table 2. Although far from industrial application, it can be concluded that majority of MXene composites present superior performance as compare to their pristine materials. These fascinating results shed a light on further exploring MCs as function-specific electronic device materials as with improved performances. 

## 5. Conclusions and Outlooks

In summary, we demonstrated recent progress in the synthesis, structural preparation, hybridization, energy and environmental applications of MC-based 2D and 3D nanomaterials. MCs have excellent mechanics, magnetism, electronic properties, and distinct 2D layered structure with adjustable components and abundant functional groups on the surface, and therefore exhibited promising applications in various fields. By properly controlling and adjusting the structure and components of MC-based materials, 2D and 3D hybrid materials with excellent properties could be obtained. 

However, compared to other 2D materials such as graphene and TMD/TMO materials, the extended applications of MCs have some limitations, and more efforts should be done in the future. First, the price of MCs is relatively high and not easy to synthesize, which inhibited their applications in practical applications. It is necessary to develop highly effective strategies to synthesize MCs with unique structure and controlled physical and chemical properties. Second, the preparation of MCs usually needed toxic chemicals in the synthesis process, which in some ways caused environmental problems. To overcome this problem, it is better to utilize green methods to prepare MCs. For this aim, simple and environmentally friendly hydrothermal methods through MC precursors could be a potential solution. Third, the development of surface modification methods for improving the stability, dispersity, and biocompatibility of MCs should be carried out. The addition of small molecules and biomacromolecules to MCs could be a potential way to achieve this aim. For instance, the conjugation of functional biomolecules such as DNA, proteins, peptides, and even self-assembled bio-nanostructure with MCs will extend their applications in biosensing and biomedical fields. Fourth, single atoms could be considered to create on the surface of MC materials to improve catalytic performance of MC-based hybrid materials via synergistic effects and single atomic catalysis, which will promote the fabrication of high-performance batteries and supercapacitors. For instance, single metal atoms like Pt, Ru, Zn, and others could be anchored onto the 2D surface of MCs to enhance their applications in energy storage devices and electrochemical catalysis materials. Fifth, deeper studies on the fabrication of 2D and 3D MC-based materials for environmental applications should be done. For instance, the effects of porous structure and surface properties of materials on the adsorption and removal of contaminations, as well as corresponding mechanisms should be investigated. Last but not the least, while MCs and their composites have exhibited superior features as energy storage and electromagnetic shielding materials, more efforts should be focused on further improving the specific performances, i.e., mechanical strength, flexibility, electrical and chemical activity, as well as cycle stability and lifetime, of the electronic devices that employs MCs, for instance, the incorporation of functional materials and/or the exploration of novel synthetic approaches, making them competitive or even replaceable to those conventional inorganic/organic materials.

## Figures and Tables

**Table 1:** Summary on various fabrication methods of MC MXenes.

Fabrication Methods	Advantages and Drawbacks	MC MXenes	Challenges	Ref.
CVD	Advantages: (1) large lateral size (2) Extremely low defect concentration Drawbacks: (1) Low efficiency (2) Complicated process	Mo_2_C and WC	How to change the experimental conditions, such as temperature and CVD growth time, to adjust the size and thickness of the two-dimensional α-Mo_2_C crystal.	[34]
		TaC	Control CVD growth time and temperature to form a good interface between the ultra-thin TaC material and the substrate, thereby significantly improving the protective properties such as oxidation and wear.	[35]
HF etching	Advantages: (1) High selectivity (2) Acceptable yield Drawbacks: (1) Highly corrosive (2) Defects in the final product	Ti_3_C_2_	Control the morphology, particle size, surface termination groups, defect concentration and yield of MXenes; pay attention to the MAX phase and etching conditions (HF concentration, etching time and temperature).	[42]
		V_2_C	To etch V_2_AlC using a mixture of lithium fluoride and hydrochloric acid; to eliminate by-products to synthesize higher purity V_2_C.	[43]
		Ti_2_C	To tailor the surface functional groups of the MXene 2D layer and use itself as another variable to adjust its performance such as wettability, electrical and electrochemical performance.	[38]
Molten salt method	Advantages: (1) Improve the chemical safety of the experimental process (2) Reduce the difficulty and cost of waste liquid treatment Drawbacks: (1) The formation mechanism of Cl-MXene is unclear (2) More etching time and higher reaction temperature.	Ti_2_C	To optimize the molar amount of molten ZnCl_2_ to promote the formation of Cl-MXenes, since the molar ratio of Al-MAX/ZnCl_2_ is a key factor in determining the final product.	[48]
		Ti_3_C_2_	Expand the range of MXene prepared by the Lewis acid etching route, and continue to study how to customize the surface chemistry and performance of Ti_3_C_2_ MXene material by changing the test parameters during the experiment.	[39]
Electrochemical etching	Advantages: (1) With high efficiency, and high yield (2) Safety and low cost Drawbacks: Excessive etching will destroy the MXene structure	Cr_2_C and V_2_C	How to optimize the etching voltage to use Cr_2_C and V_2_C as effective electrocatalysts for oxygen release reaction and hydrogen release reaction	[33]
		Ti_3_C_2_	By optimizing the electrochemical etching parameters to reduce the production of carbon derivatives, high-quality Ti_3_C_2_ can be obtained	[40]
Hydrothermal method	Advantages (1) Large interlayer distance and large specific surface area (2) Environmental- friendly Drawbacks: (1) Easy to form by-products, such as aluminum hydroxide (2) Few studies on the formation mechanism of crystal nuclei and high dependence on equipment	Ti_3_C_2_	How to control the temperature and alkali concentration to remove aluminum compounds, promoting the entire etching process to the production of MC MXene.	[41]
		Nb_2_C	Control the temperature and hydrothermal time to form a slow-release mechanism in the hydrothermal process to obtain Nb_2_C materials with higher c lattice parameters, greater distance between layers and greater BET specific surface area.	[54]

**Table 2:** Typical electronic devices that employ MXene-materials with different functions and their performances.

Electronic Devices	MXene-Materials	Functions	Performance	Ref.
SIBS	Ti_3_C_2_/NiCoP	Energy storage	Unique synergy between NiCoP nanoparticles and Ti3C2 framework, the as-prepared Ti3C2/NiCoP electrode exhibits reversible capacity, high rate performance and excellent cycle performance.	[72]
Supercapacitors	V_2_C	Energy storage	Excellent specific capacitances were achieved, specifically 487F/g in 1M H_2_SO_4_, 225F/g in 1M MgSO_4_, and 184F/g in 1M KOH, which are higher than previously reported values for few micrometer-thick delaminated MXene electrodes.	[74]
LIBs	Mo_2_C	Energy storage	As the anode material of rechargeable batteries, the synthesized Mo_2_C MXene has a capacity of 150 mAh g^−1^ for LIBs, showing excellent rate performance and cycle stability.	[75]
Battery-like SCs	CoF/MXene	Energy storage	The maximum specific capacitance of CoF NPs, MXene, and CoF/MXene composites was observed to be about 594, 1046.25, and 1268.75 Fg^–1^ at 1 A g^–1^, respectively. The calculated specific capacity of the CoF/MXene composite was about 440 Cg^–1^ at 1 A g^–1^ and proved to be an excellent hybrid electrode material by providing only 0.25 Ω charge transfer resistance. The as-synthesized material demonstrated the excellent capacitance retention, about 97%, up to 5000 cycles.	[76]
Spercapacitors	Ti_3_C_2_Tx-K	Energy storage	The electrode prepared from the K+ assisted self-assembly of Ti_3_C_2_T_x_ nanosheets (Ti_3_C_2_T_x_-K) exhibits an excellent capacitance up to 1025 mF cm^2^ (427 Fg^−1^) at a scan rate of 2 mV s^−1^ and impressive rate capability and cycle stability.	[77]
Stealth devices	Ni@Ti_3_C_2_	Electromagnetic Wave-Absorbing	Excellent impedance matching, magnetic coupling effect, conductance, magnetic lose and multiple scattering contribute to the excellent electromagnetic wave absorption performance of the Ni@MXene hybrid. By combining the excellent dielectric properties of (Polyvinylidene Fluoride) PVDF, the EM wave-absorbing performance of the Ni@MXene hybrid is developed to a great extent. The PVDF composite with 10 wt% Ni@MXene 8:1 hybrid shows relatively hunk absorbing properties with an RLmin of −52.6 dB at 3 mm and optimal EAB of 6.1 GHz. By adjusting the sample thickness, the EAB of all of the hybrid composites can cover completely the whole X-band.	[58]
Wearable radio-frequency wireless communicators	Ti_3_C_2_T_x_-SWNT	Electromagnetic interference shielding	The resulting MXene-single-walled carbon nanotubes (S-MXene)/latex device was able to sustain up to 800% areal strain and demonstrated strain-insensitive resistance profiles for 500 stretching/relaxation cycles, which encouraged its applications in stretchable EMI shielding and wireless communicators. A layer of stretchable S-MXene conductor demonstrated strain-invariant EMI shielding performance ≈30 dB under wide ranges of areal strains, and the shielding performance was improved to 47 and 52 dB by stacking 5 and 10 layers of S-MXene conductors, respectively.	[100]
Next-generation smart devices	Ti_3_C_2_/Graphene	Electromagnetic interference shielding	The EMI SE of Mxene can reach 50.7 dB, which is higher than the EMI SE of reduced GO foam. Mxene’s high conductivity and lightweight characteristics make it have excellent specific shielding effectiveness (6217 cm^3^ g^−1^)	[102]
Ultra-thin electromagnetic shielding coating	TiC_2_Tx/PEDOT:PSS	Electromagnetic interference shielding	The thickness of TiC_2_Tx/PEDOT:PSS is only 11.1 μm, but it shows a high EMI SE value of 42.10 dB. Compared with pure Ti_3_C_2_Tx MXene film, the tensile strength increased from 5.62 MPa to 13.71 MPa, and the fracture strain increased from 0.18% to 0.29%.	[104]

## Data Availability

Not applicable.
